# Crises information dissemination through social media in the UK and Saudi Arabia: A linguistic perspective

**DOI:** 10.1371/journal.pone.0284857

**Published:** 2023-05-05

**Authors:** Rukayah Alhedayani, Reem Alkhammash

**Affiliations:** 1 Department of the English Language, College of Language Sciences, King Saud University, Riyadh, Saudi Arabia; 2 English Department, University College, Taraba, Taif University, Taif, Saudi Arabia; Tallinn University: Tallinna Ulikool, ESTONIA

## Abstract

This study investigates health-promoting messages in British and Saudi officials’ social-media discourse during the Coronavirus Disease 2019 (COVID-19) Pandemic. Taking discourse as a constructivist conception, we examined the crisis-response strategies employed by these officials on social media, and the role of such strategies in promoting healthy behaviors and compliance with health regulations. The study presents a corpus-assisted discourse analysis of the tweets of a Saudi health official and a British health official that focuses on keyness, speech acts, and metaphor. We found that both officials utilized clear communication and persuasive rhetorical tactics to convey the procedures suggested by the World Health Organization. However, there were some differences in how the two officials used speech acts and metaphors to achieve their goals. The British official used empathy as the primary communication strategy, while the Saudi official emphasized health literacy. The British official also used conflict-based metaphors such as war and gaming, whereas the Saudi official used metaphors that reflected life as a journey interrupted by the pandemic. Despite these differences, both officials utilized directive speech acts to tell audiences the procedures they should follow to achieve the desired conclusion of healing patients and ending the pandemic. In addition, rhetorical questions and assertions were used to direct people to perform certain behaviors favored. Interestingly, the discourse used by both officials contained characteristics of both health communication and political discourse. War metaphors, which were utilized by the British Health official, are a common feature in political discourse as well as in health-care discourse. Overall, this study highlights the importance of effective communication strategies in promoting healthy behaviors and compliance with health regulations during a pandemic. By analyzing the discourse of health officials on social media, we can gain insights into the strategies employed to manage a crisis and effectively communicate with the public.

## Introduction

Although social media offers a great deal of content exposure and engagement with users, managing health-related crises through Twitter, for example, can be rather difficult. In particular, it can be difficult for Health Officials (HOs) to use this social-media service to change or influence the public’s perceptions [1]. Many communication researchers view social media as having both benefits and disadvantages in times of crisis [2–4]. Regarding disadvantages, for many officials managing crises through Twitter is relatively unfamiliar, as there are few available guidelines for handling crises through social media [5]. Further, another challenge concerning effectively disseminating health-related messages through Twitter is widespread health misinformation; this was particularly notable in the context of Coronavirus Disease 2019 (COVID-19) and its vaccines. During the COVID-19 Pandemic, an unprecedented amount of misinformation flooded social media, resulting in an infordemic [6]. This included theories concerning the origin of the virus and claims about home remedies that could cure the disease; such theories may have contributed to vaccine hesitancy and opposition to vaccines. Officials are often viewed as key figures for not only providing the most accurate information, but also for changing misperceptions among the public. Thus, it was imperative for officials to use their social media accounts to debunk false information and advocate for informed health decisions; for example, by confronting anti-vaccine campaigns. To achieve desirable outcomes, HOs and governments sometimes apply strategies such as coercion, persuasion, and directing emotional responses [7].

The COVID-19 Pandemic created a new global crisis, which led to a coalition between politicians and HOs to enforce novel health measures. Consequently, a previously under-investigated behavior became prominent: political speeches on health topics. There have been many studies of this behavior since the beginning of the pandemic. For example, transparency and legitimacy are useful strategies to help preserve people’s health and well-being [6], and some research argues that strategies employed by the Pakistani government helped in reducing the number of people who contracted the disease [8]. Additionally, Semino [9] investigated the metaphors used by politicians and the media when discussing COVID-19. She consequently found war metaphors to be common, and suggested that substitute metaphors should be used in their place. Meanwhile, Alyeksyeyeva et al. [10] investigated the rhetoric of the Australian prime minister regarding COVID-19. They argued that:

[r]hetoric plays an influential part in managing crisis, therefore political leaders frame the disease outbreak in war imagery and ‘fear-language’ to get the public involved in what they call ‘national interest’ and make the audience accept the leaders’ decisions, which enhances their authority

(p.92).

Rhetoric, therefore, plays an important role in directing public opinion and reducing crisis effects on the public and private sectors as well [11]. In addition to rhetoric, the framing of ideas can represent “a matter of life and death” [12]. Maani et al. [12] argue that the manner by which officials frame a problem to the public can lead to support for certain therapies in place of other, far more urgent, therapies.

Some tweets can be explicitly identified as formal requests or appeals to the public to perform specific actions. However, such tweets do not include any consideration of people’s agency regarding the actions in question. Recipients of the tweets are presented with a single solution, and are continuously directed to adhere to this recommendation or appeal (in the context of COVID-19, such recommendations/appeals are generally from health organizations or HOs). Therefore, we felt that consideration of speech acts would represent a good starting point for the present study, which focused on HOs’ COVID-19-related discourse on Twitter; this was because through analyzing such acts we could determine how HOs communicate to the public and seek to direct them towards desirable outcomes.

The specific objective of the present study is to reveal how HOs from different cultural contexts differ linguistically in terms of their use of lexical items and rhetorical devices when tweeting crisis communication regarding the COVID-19 Pandemic. Specifically, this study sets its main research question as follows: what crisis-response communication strategies have been employed by HOs during unprecedented health crises? To achieve this objective, we investigated how HOs in Britain and Saudi Arabia used social media to assist their management of the pandemic, and how they urged the public to conform to the guidelines of the World Health Organization (WHO). We gathered tweets by both health officials within the time frame from January 2020 till March 2021. The Arabic tweets were analyzed in Arabic and later translated to English for the readers’ convenience. We analyzed their tweets from three perspectives–keyness, rhetoric, and metaphor. We begin by conducting a keyword analysis; we performed this in order to identify common topics discussed by the two HOs and to investigate similarities and differences in how they communicated these topics to the public. The second perspective involved a speech act analysis. The dataset used for this analysis comprised tweets that aimed to convince people to follow certain procedures. The officials’ argumentation style was investigated in this phase. The third perspective concerned the metaphors used by both HOs. Metaphor represents a major aspect of health communication and, thus, we felt it was necessary to include it. Metaphors influence how concepts are framed, and can affect how people respond to the content being communicated to them.

The present paper is structured as follows: the next section presents a review of related literature; section two presents the data, along with the methodology employed in their collection and analysis; section three features a discussion of findings; and section four contains concluding remarks.

## Literature review

The first angle from which this paper considers the data is the rhetoric use of speech acts by HOs to persuade the public to follow recommended steps. Speech Act Theory, which was first introduced by Austin [13] and further developed by Searle [14, 15], pertains to how utterances can comprise locutionary acts, illocutionary acts, and perlocutionary acts. Locutioanry acts refer to what is meant, illocutionary acts refer to what is done, and prelocutionary acts refer to what happens as a result of what is said [13]. According to Searle [15], there are five types of illocutionary speech acts: assertive, such as informing and claiming; commissive, such as promising and swearing; directive, such as ordering, requesting and advice; declaratory, such as naming and dismissing; and expressive, such as complaining and congratulating. With later developments in Speech Act Theory, however, further acts were added, such as apology, wishes, and gratitude [16]. Searle [15] also differentiated between direct (literal) and indirect speech acts. In indirect speech acts a sentence form is used to convey a function for which it is not conventionally used [17]. For example, an interrogative form is conventionally used for asking questions, but can be used indirectly for requests as in the sentence *Could you turn out the lights*? (example is from [17]).

Speech Act Theory focuses on how speakers use words to perform certain actions, and on the effect these words have on the listener. We follow the taxonomy of speech acts as presented by Searle [15] to categorize the speech acts we find in the tweets corpus. We also adopt further classifications found in [18, 19]. Table 1 shows the speech act taxonomy adopted here.

**Table 1:** Taxonomy of speech acts.

Speech Act	Function
Assertive	Statement of facts, getting the receiver to form or attend to a belief
Directive	Getting the receiver to do something
Commissive	The HO commits themselves to do something
Expressive:	Expression of feelings or knowledge
Thanks	
Apologies	
Wishes	
Greetings	
Declaration	Change in institutional state of affairs
Interrogative	Asking questions

Taxonomy is adapted from Nastri et al. [18] and Elliott-Maksymouicz et al. [19] and is based on Searle [15].

We chose to consider the speech acts used by the two HOs in their tweets in order to determine the argumentation styles they used to lead their respective audiences towards adherence to the WHO guidelines. Argumentation on Twitter has previously been investigated by Elliott-Maksymowicz et al. [19], who found that Twitter-based arguments are often of a very simple nature; such arguments generally comprise a specific form of qualitative content that is defined in a series of logically connected assertions that result deductively in a conclusion. They were also able to show that several different types of speech acts (not just assertions) can be utilized to make argumentative points. Our investigation sheds light on the HOs’ use of social media, especially Twitter, in order to communicate health advice and directions for the public to guide them in time of crises. Therefore, we predict that the speech act of directives is used frequently in the tweets of HOs. However, leaders do not only direct people to what to do, they also try to lift their spirits (expressive speech acts), pass laws (declaration speech acts), and announce future plans (commissive speech acts). This study investigates the different speech acts found in the HOs’ tweets, and attempts an explanation for the ones not found there.

The second angle we investigate is metaphors used in the data. Metaphors shape thinking and, in turn, language shapes how people think of a certain subject [20]. Metaphor is defined as the use of language to discuss a certain thing in terms of something else. The two things in question are mostly different, but some similarities can be identified between them. These similarities are highlighted through metaphor. For example, when someone says “this is heaven” while eating a piece of cake, they highlight a similarity between the two, possibly *pleasure*. Metaphors appear frequently in language. Some research suggests that metaphors occur 3–18 times per 100 words [21, 22]. In addition to their frequency, metaphors are said to have a crucial role as a communication tool. Conceptional metaphor theory [20] posits that conventional metaphoric expressions can be used to propose the existence of conceptional metaphors, where conceptional metaphors refer to systematic mappings between a target domain and a source domain. For example, the expression h*e was filled with anger* contains the conceptual metaphor that “the body is a container.” Lakoff and Johnson [20] also state that conceptional metaphors are used to facilitate certain inferences and evaluations, which makes them important communication tools for explanation or persuasion.

In terms of health-related communication, studies, particularly those focusing on AIDS, have shown the potency of language to shape the impact of epidemic disease [23]. Wallis and Nerlich [23] found that war metaphors were absent from UK media coverage of the 2003 SARS epidemic, but prevalent in discussions of cancer. However, the use of military metaphors in discussions of illnesses has been widely criticized. For example, Sontag [24] states that such metaphors promote shame and guilt among patients, in addition to an excessive desire among policy-makers and politicians to exercise control over the disease. Therefore, there have been wide calls to remove all metaphors from health communication [24], or to replace them with “liberating” metaphors [25]. Semino [9], for example, suggests the use of “firefighting” metaphors for COVID-19 prevention and cure.

This extensive body of literature on metaphor in English is counteracted by a shortage of research on metaphor in Arabic. In general, research on Arabic health communication is scarce [26]. The present study, therefore, represents a step towards bridging this gap.

## Data and methods

### Corpus compilation

This section comprises a description of the data used and of the methodology followed in the present study’s analysis. The data investigated in this study were taken from the Twitter accounts of a HO in Saudi Arabia and a HO in the UK. The collection and analysis method followed in this paper complies with the terms and conditions of Twitter. Twitter provides a platform for users to write short blogs and incorporate visual media with their text. Both officials added visuals to some of their tweets. Although we did not conduct a multimodal analysis of the visual data contained in the officials’ tweets, we nevertheless used the visual data to guide our interpretation of the tweets.

To address the research question, a specialized corpus was compiled: the “Arabic and English health official online corpus” (AEHOO) which is used specifically for the purposes of this paper. The tweets were collected from the British and Saudi officials’ Twitter accounts for the period from January 2020 to the end of March 2021. The British HO at that time was Nadine Dorries (female) and the Saudi HO was Tawfiq Alrabiah (male). We did not observe any major differences regarding gender, so this variable was ignored. This is in line with previous literature who analyzed leaders’ speeches during COVID-19 and have not noticed gender as a crisis communication strategy variable (See [27]).

During the period of data collection, we gathered 86 tweets from the Saudi HO (hereafter, “SHO”; six tweets were excluded because they were in English), and 200 from the British HO (hereafter, “BHO”). The English sub-corpus of the AEHOO contained 6,207 words and several hashtags, such as “#NHS,” “#covid,” “#Patientsafety,” “#CovidVaccination,” “#StayHome,” and “#Lockdown,” while the Arabic sub-corpus contained 3,677 words and hashtags such as “#weareallresponsible,” “#takethestep,” and “#coronavirus”. These hashtags were tweeted in Arabic. They have been translated here by the authors for the readers’ convenience. There is a notable difference in the number of words between the two sub-corpora. However, we do not have reason to believe that this can affect the results. We chose to constrain our data collection using a time frame rather than by number of words because this helps to uncover the different way both HOs employ their twitter accounts which serves the objective of this paper. The Twitter data were gathered manually, and were cleaned and converted into plaintext before the corpus was uploaded to Sketch Engine [28]; Sketch Engine is a user-friendly online platform that allows searches for linguistic patterns using corpus-based tools such as keywords, collocation, and concordance [29]. By integrating the two sub-corpora, the AEHOO included tweets from both the BHO and SHO.

### Sketch engine: An online platform for processing multilingual corpora

This study featured a bottom-up investigation of keywords in the AEHOO; this differs from top-down analysis, which requires *a priori* assumptions about a discourse’s lexical components [30]. In our bottom-up analysis, we performed corpus-assisted discourse study, which involves comparing a corpus to a reference corpus to highlight relevant terms [31]. In particular, keyword analysis was used in addition to the creation of conventional frequency lists [32].

The keyword function of Sketch Engine was used to identify the keywords in the two sub-corpora. Furthermore, as both English web 2020 (enTenTen20) and Arabic web 2012 (arTenTen12), two standardized web text corpora, are available in Sketch Engine and have fundamental genre-related qualities, we used both as general reference corpora to yield keyword lists. The statistical significance of the identified keywords was determined using the logDice test, which ensured that their keyness was not due to random chance [31]. These keywords were then semantically classified and tabulated. A keyword score of 100 was used as a cut-off point, and all words of that score and above were copied to an Excel sheet. Thirty-five keywords were identified in the English corpus and 138 in the Arabic one. There are two reasons for the difference in the number of keywords. First, the reference corpus used for the Arabic data was an excerpt of the arTenTen, which is not continuously updated, while the reference corpus for the English corpus is continuously updated; this increased the likelihood of the two English corpora being more similar. Second, linguistic idiosyncrasies of Arabic mean the same word might be used in numerous different forms. An example is the word “infected,” which appears in the keyword list for the Arabic corpus in three word forms; another example is the word “precautionary,” which appears in four word forms.

After this stage, insights obtained from linguistic analytical approaches such as rhetorical analysis and metaphor analysis were applied based on the framework presented in the literature review.

## Results

To obtain an adequate understanding of how the two HOs managed the COVID-19 pandemic, how they discussed it and presented it to their respective publics, and how they guided the people to perform desired behaviors, we applied a three-step process: we first conducted a lexical analysis to identify the common topics discussed by both HOs; the lexical analysis is presented in the following section. Second, we investigated the speech acts used in both corpora. The rationale behind exploring the speech acts was that these tweets were intended to educate the public about a new disease. The HOs wanted the public to perform certain behaviors in order to prevent the spread of this new disease. They wanted the public to act, so they communicated to them how they themselves were acting to combat the virus, and explicitly directed them through their language use. Therefore, Speech Act Theory lends itself well to this type of discourse. Finally, we discuss the metaphors used by both HOs when discussing the pandemic.

### Keyword analysis

By classifying nouns semantically, we obtained the following results. First, because both officials talk about the same topic, both the BHO and the SHO communicated the same general messages regarding managing COVID-19, such as naming the virus and advocating the public adoption of necessary measures to minimize its spread. However, we did observe some divergences in focus between the two, which might reflect the different management strategies implemented in the two countries (see Tables 2 and 3). The BHO addressed specific issues relating to certain events, people, or places, while the SHO spoke more generally about health care and medical support for tackling the virus. Further, the BHO mentioned specific medical equipment and medical conditions and gave specific numbers regarding the financial requirements of projects developed to combat the pandemic, while the SHO did not tweet any such specificities, with the exception of his mentioning of Nujood Alkhaibari, a Saudi nurse who passed away as a result of COVID-19. Finally, attention to specific COVID-19-related strategies was more evident in the BHO’s tweets, with the BHO highlighting the emotional support that should be implemented to help others manage losses caused by COVID-19.

**Table 2:** Semantic categorization of keywords in the English corpus.

Semantic category	Keywords
COVID-19	Covid-19, coronavirus, covid, covid19
Precautionary measures	Lockdown, distancing
Vaccination	Biontech, Pfizer, immunization, rollout
People	Jvt, scouser, scouse, BAME, grandad, dph, moira, zuzan
Places	Leicester, Midbed
Handling loss	Bereavement
Financing projects	pound300, pound3m, pound24, pound5m, pound27m
Facility shortages	hospitalisations-lag
Medical condition	RTA
Medical equipment	PPE

Source: data gathered by the authors.

**Table 3:** Semantic categorization of keywords in the Arabic corpus.

Semantic category	Keywords
COVID-19	Virus, corona, covid-19, new, pandemic
Precautionary measures	Protocols, precautionary, handshake, lockdown, face mask, sterilization, crowded, wear, hands
Vaccination	Vaccine, safety, dose
People	Alkhaibari, Nujood
Places	Wuhan
Symptoms	Pass away, hurt, sneeze, cough
Infection	Contracting, infection, transmit
Health care	Volunteer, clinics, practitioner, swap, readiness, prescriptions, hospitalization, medical, pharmacy

Source: data gathered by the authors.

### Rhetorical analysis

This section discusses the speech acts used by both HOs. This is considered a suitable analysis approach for the current study because Speech Act Theory has been previously used to investigate both health-care communication [33, 34] and political discourse [19, 35]. The corpus analyzed in the present paper comprises tweets by HOs that are directed at the public. It is true that the tweets did not comprise two-way conversations; however, there were some features that caused these tweets to represent conversation. For example, the data included interrogative forms and imperative forms, which can be used in conversation. Also, people can retweet and comment on tweets which can be regarded as two-way conversation. Further, contextualization of ideas was also present. When the HOs discussed a topic, they expected their audience to already be familiar with content they had mentioned previously. Examples of this contextualization appeared on a number of occasions, and was mainly represented through emojis and photos. The following tweet by the BHO is a good example of this:

(1) That’s me done. (syringe emoji)

Tweet (1) above was accompanied by a photo of the BHO receiving a COVID-19 vaccine. Both the emoji and the photo served as contextualization cues that helped the audience understand the text of the tweet. However, contextualization is beyond the scope of this paper and, therefore, will not be discussed further. In contrast, questions and imperatives are within our speech act analysis because they can be used (directly or indirectly) to preform directive and/or interrogative speech acts. Table 4 shows the list of speech acts used in both HOs’ tweets.

**Table 4:** Frequencies and percentages of speech acts in each corpus.

Speech act	BHO (200 tweets)	%	SHO (80 tweets)	%
**Assertion**	131	55.7	45	32.4
**Directive**				
**direct**	20	8.5	34	24.5
**indirect**	14	6	20	14.4
**Declaration**	0	0	0	0
**Expressive**	17	7.2	0	0
**Thanks**	17	7.2	16	11.5
**Wishes**	4	1.7	13	9.4
**Greetings**	3	1.3	0	0
**Apologies**	0	0	0	0
**Interrogative**	16	6.8	1	0.6
**Commissive**				
**direct**	11	4.7	1	0.6
**indirect**	2	0.9	9	6.6
**Total (sentences)**	235		139	

Source: data gathered by the authors. BHO: British health official; SHO: Saudi health official

The assertion speech act was prominent in both data sets, as Table 4 shows. The frequencies of the other speech acts varied across the two corpora. Speech acts are counted based on sentences not tweets. Therefore, the number of speech acts in this table is greater than the number of tweets. The different speech acts are discussed below in detail.

#### Assertion

Most of the tweets in our corpus were assertions. The strength of assertions is that the content being communicated is not only intended to inform the recipient, but also features an expectation that they will believe this information as true since it comes from a health official. While the “social” aspect of assertions has been challenged by Pagin [36, 37], we follow the theory of Marsili and Green [38], who state that assertions can be social speech acts. Specifically, Marsili and Green [38] argue that assertions are similar to other speech acts because they represent a social phenomenon that can only be understood by knowing the situation in which they are uttered and who the speaker and recipient are. Thus, the present work considers assertions by the HOs to be social acts because, by knowing the authors of the tweets (HOs), the audience was inclined to take them more seriously. Health information should be obtained from reliable sources, and HOs represent reliable sources for the public. Therefore, in our context, assertions represent a social act in which the HOs communicate information to the public and expect the public to believe this information and reject contrasting information from other sources. Examples of assertions are listed below.

(2) My responsibility towards my country is important; therefore, I will keep adhering–God willing–to the precautionary measures to safeguard others from infection. (SHO)
(3) All vaccines used in the Kingdom show high protection against the virus two weeks after administration, in addition to high safety. (SHO)

Tweet (2) is accompanied by a drawing of a Saudi person to show that it is not the HO speaking. In this tweet, the SHO presents an argument intended to persuade the public. This argument contains an assertion featuring a first-person subject, which is intended to have the reader consciously or unconsciously adopt the argument as their own. The SHO asserts that one’s responsibility towards their country requires him/her to adhere to the precautionary measures recommended by their country’s government. This assertion asks the public to believe in this motto and adopt it as a measure to prevent infection. Tweet (3) is also an assertion, and is intended to reassure the public that vaccines are effective. This tweet features an expectation that the public will obtain vaccines as a result of this reassurance. The argumentative property of this tweet is more visible than that contained in Tweet (2). No quantitative estimate of safety evidence, such as exact numbers, was presented by the SHO. Instead, the SHO focused on health literacy as a communication strategy, as he only reported a qualitative estimate of safety; in this case, simple frequency (see [39, 40] for the difference between quantitative versus qalitative estimates of risks and safety).

Similar tweets can also be found in BHO dataset. Consider the following:

(4) Those who cite low daily diagnosed #COVID19 cases as a reason to exit lockdown now, miss the point. They are low, because of lockdown. (BHO)
(5) My 84yo mum couldn’t have been happier to be called up for her #CovidVaccine today. (BHO)

In tweet (4), the BHO corrects what she views as a misconception by people who are advocating an easing of lockdown measures. She explains that they were “miss[ing] the point” and, therefore, that the public should not believe them. In tweet (5), she asserts her mother’s state regarding taking the vaccine, which is part of an effort to encourage others to do the same. By mentioning a personal anecdote in a public discourse regarding health promotion (5), the BHO uses empathy as a communication strategy; this strategy is generally used to enhance credibility and foster effective communication [41].

#### Expressive

Expressive content reflects feelings and epistemic knowledge [19]. According to Searle [15], expressive speech acts include apologies, thanks, whishes, and greetings. However, because expressive speech acts tend to be highly routinized [42], genuine ones are distinguished from ritual phrases. However, Weigand [43] considered both as speech acts. In her taxonomy, sincere thanks and apologies are included under *emotives* and when they are used as formulaic expressions, they are included under *declaratives*. This dual role of expressive speech acts is supported by the use of thanks and apologies in Covid-19 signage [34].

In this category, we include tweets containing verbs such as “know” and “love” in such content. These expressives constituted 7.3% of the tweets by the BHO and 0% of the tweets by the SHO. It is not surprising that this category represented such a small proportion of the tweets, as the HOs’ tweets had a general aim of educating the public about a new disease, and personal feelings were of secondary importance. The tweets that expressed epistemic knowledge generally followed the theme of “we do not know much about this disease.” Sentence (6) provides an example from this category in which an act performed by the BHO that fosters a certain emotion is expressed.

(6) We learn more about this virus every day. (BHO)
(7) It is such a huge privilege and pleasure to work with local authorities in the development of their local outbreak management plans. (BHO)

On the other hand, we did not find any expressive tweets from the SHO; he did not mention any mental or emotional acts at all. Thus, there was a divergence between the BHO and the SHO in this regard; the BHO seemed to show more emotions than the SHO. This may partly reflect gender differences (the BHO was female while the SHO was male), and also indicate that the BHO applied a communication strategy of using emotion to show empathy (see [44]).

Expressive speech acts also include thanks, apologies, greetings and wishes. In this data, there are no apology speech acts. Both ministers emphasize that they are following the best guidelines the present situation requires. The other speech acts, however, appear in varying frequencies.

#### Greetings

Greetings appear only three times in the BHO tweets. One difference between the two HOs is that the BHO interacts with people who praise or criticize her work. She quotes their tweets, greets them, and replies to what they say.

#### Thanks

Both HOs regularly made “thank you” remarks or similar expressions. Thanking expressions are reactive speech acts, which means they are a response to a past act by another [45]. These expressions can generate solidarity between interlocutors or, as in our case, between HOs and the public or other members of the health-care system.

(8) I thank all government sectors that acted quickly to elevate levels of readiness and complementarity, that secured information for travelers and flights, and that activated precautionary measures at land borders, seaports, and airports to protect our country from this virus. (SHO)
(9) Thank you Sean, and your team for your collaborative approach and your determination to beat #Covid in Oldham. (BHO)
(10) Never all those years ago could I have imagined what our nurses today have to deal with on a day to day basis. We are so grateful, thank you. (BHO)

The above tweets show expressive thanking speech acts in which the HOs thank certain people for performing acts that helped slow the spread of the virus. However, thanking can also occur in advance, before an act is performed, such as when thanking people for their compliance with precautionary measures; this form of thanking can be seen in tweet (11).

(11) Thank you for your commitment to wearing masks when out of your homes. (SHO)

In this tweet, the SHO thanks the people for their commitment to wearing face coverings; this represents an encouragement to the public to continue following this advice. This, and similar instances, however, are counted as indirect directive speech acts because their purpose is to direct the people to do something rather than thanking them for something they did.

#### Wishes

The final category of expressive speech acts found in the two corpora is wishes. The optative mood appeared regularly in the SHO’s tweets (in 9.4% of the total tweets in the corpus). These tweets expressed a prayer or a wish. Meanwhile, there were only four tweets of this type in the BHO’s sub-corpus; two of these tweets involved the HO saying “good luck” to someone. The other two are listed below:

(12) I wish @realDonaldTrump a good recovery from this ghastly #Covid virus.
(13) Best wishes to all Alevel students in MidBeds and everywhere.

In tweet (12), the BHO expresses a wish that the President of the United States of America will have a good recovery from COVID-19. In tweet (13), she provides encouragement to students who are taking exams during the crisis.

The Arabic data included thirteen tweets that could be classified as wishes. These tweets included the following:

(14) May God keep us all safe from any harm.
(15) I wish you lasting health.

Batanova [46] suggests that well-wishing is culturally determined and that it plays a pivotal role in some cultures.

#### Commissive

Tweets labeled as commissives featured promises, vows, or intentions. This category comprised 7.2% of the SHO’s tweets and 5.6% of the BHO’s tweets. In tweets (16) and (17) below, the HOs promise the people that they will act in the public’s best interests. They vow to help the people end this pandemic.

(16) Your safety and health are a priority to us. (SHO)
(17) We must do all we can to prevent our ICUs #NHS from becoming overwhelmed. (BHO)

Both the social aspect and argumentative aspect of speech acts are present in commissives. The HOs were addressing the public directly, inviting them to become active players in the attempt to overcome the crisis. The HOs were also assuring the public that they were important and that they could help. We observe here that both HOs used openness, frankness, and honesty as a communication strategy. This approach accords with recent research findings regarding the most effective communication strategies government officials can use to encourage people to perform certain behaviors during the COVID-19 Pandemic [47].

#### Declaration

Declaration speech acts bring about a change in the world. There are no instances of declaration speech acts in the data. The HOs do not use any performative verbs expressing illocutionary acts that change the state of affairs. We find the lack of declaratory acts surprising as the HO are expected to declare procedures to be followed by the public. This behavior by the HOs can be explained by their adherence to the suggestions announced by WHO. The strategy of openness and frankness as represented in commissive and declaratory speech acts appears in a small number of tweets. Declaration was not found in the tweets and commissives are used less than other speech acts such as assertions, thanking, directives, and even interrogatives (in BHO tweets). This might suggest that the HOs assumed their responsibility as leaders in persuading their respective audiences, directing them, and being empathetic with them. As for passing laws, they both followed the recommendation of WHO.

#### Directive

In a directive speech acts, orders are expressed. However, the imperative form of the verb is not the only verb type included in this category. Directives can vary in strength from mere invitations to direct orders and, thus, verbs such as “call for” and “encourage” are included in this category because, similar to the imperative, they can be used to ask the listener to act. Examples include the following:

(14) Dear brothers and sisters, be very mindful of the preventive measures, we do not want you, your parents, or loved ones to be the next casualty. (SHO)
(15) Do not forget our health-care heroes in your prayers. (SHO)
(16) I recommend for everyone to use a face covering if they need to leave the house. (SHO)
(17) …don’t touch the mask to lower it to speak over and wash after every use. (BHO)
(18) Please follow the guidance. (BHO)
(19) Be proud (British flag emoji). (BHO)

Tweet (19) was accompanied by a map that showed that Britain was the country with the highest number of vaccines administered at that point in the pandemic.

The speech act of directive is used repeatedly in the corpus. This is understandable as, to guide the public through the pandemic, the HOs were telling the people what they should do. This category comprised 14.5% of the BHO’s tweets and 38.9% of the SHO’s tweets. In these tweets, both HOs applied clear communication as their main communication strategy; this was in order to maintain order and reduce anxiety by emphasizing concrete action [47]. However, there is a large difference between the way the SHO uses directives and the way the BHO uses them. First, the SHO uses directives more than the BHO. Even though the number of words in the Arabic tweets was smaller, the SHO used more directives than the BHO both directly and indirectly. This indicates a divergence in their communication strategies. The SHO used 34 direct directive speech acts while the BHO used 20 speech acts. This might indicate that the SHO views guidance of the public as his main goal while the BHO focuses more on supporting the public and values empathy. There are also instances of indirect directive speech acts. In these speech acts, the HOs use declarative statements as directives Examples include the following:

(20) Important and urgent: If you suspect that you have Covid symptoms, you can easily check your health status using “personal assessment” in the app Mawid. (SHO)
(21) We have the capacity and the ability, but we need people to come forward for testing in order to do the tests

In these tweets, the HOs reduce the force of the directive by not using direct imperative forms to mitigate the effect they might have on the public.

#### Interrogative

An interrogative speech act demands a reply to a question. However, interrogatives can be used in various ways; for example, as rhetorical questions or discourse markers. There was a large difference between the two HOs regarding their use of interrogative content in their tweets. That is, they used questions differently. The SHO only tweeted one question, and this was a genuine request for information.

(20) As a result of the precautionary measures, the number of seasonal flu cases has decreased by over 98% in the past three months when compared to the same period last year. What do you suggest we change in our customs to keep infection rates low once the pandemic has ended? (SHO)

Here, the SHO is inviting people to suggest ways of lowering infection rates of seasonal flu in the future; the public could respond using Twitter’s “reply” functionality. Here, the HO was again encouraging people to be active participants during the crisis; he was inviting people to think and produce solutions.

Meanwhile, the BHO did not use the interrogative structure to ask direct questions. Instead, she used it rhetorically. In political discourse, rhetorical questions are used in attempts to persuade the audience by appealing to their emotions [48].

(21) …in fight against #COVID19 Remember when we were told that [because] we had left the EU we would be at the back of the queue for vaccines? We were at the front and have secured 40m of the Pfizer vac (BHO).
(22) It’s over 60s who are at risk. How do you shield 13 m people? (BHO)

In tweet (21), the interrogative is not used to question, but rather to remind the reader of an incident that occurred sometime before. This question is used as a discourse marker. The BHO wants to refute an argument and, therefore, foregrounds it to ensure that the reader knows what she is talking about.

The interrogative in tweet (22) is used to appeal to the public’s emotions. She wants them to appreciate the gravity of the situation by asking them to think about a difficulty that she faces as a HO. The use of interrogatives as a rhetorical tactic serves an important function as an effective persuasive device (see, for example, [49]).

The speech acts discussed in this section reflect the role each HO assumed. By conceptualizing the HOs’ language as actions, we were able to identify the patterns of persuasion and argumentation used by both. On some occasions, the HOs used direct imperative acts to tell the public what to do, on other occasions they appealed to their emotions by asking rhetorical questions, and on still more occasions they built solidarity with their respective audiences by using wishing and thanking acts. The speech act analysis also reflected the modes of communication employed by the two HOs. Although they applied generally similar acts, they utilized these acts differently. The BHO communicated her emotions more frequently than did the SHO. She also appealed to the emotions of her audience more frequently than he did. This clearly shows how the BHO used empathy as a communication strategy. On the other hand, the SHO acted as a channel for promoting health literacy among the public.

### Metaphor

This section focuses on metaphorical references to the COVID-19 Pandemic in the HOs’ tweets. Analysis of metaphor use can identify how certain issues are viewed and are, in turn, framed. Metaphor use in health communication has been discussed by many researchers [24, 50, 51], who found that war metaphors are used frequently in this type of discourse. In war metaphors, diseases are perceived as an enemy, and patients are said to be fighting against the enemy. Thus, people talk of battles in which they/patients/the public must fight/defend against an enemy. This metaphor type was found in the BHO’s tweets, albeit rarely. Tweets with metaphoric references to the pandemic represented just 4% (eight tweets) of her entire corpus. Examples of these eight metaphoric references are listed below.

(27) In fight against #COVID19 Remember when we were told that [because] we had left the EU we would be at the back of the queue for vaccines? We were at the front and have secured 40m of the Pfizer vac [syringe emoji].
(28) Thank you Sean, and your team for your collaborative approach and your determination to beat #Covid in Oldham.
(29) Just finished a zoom call with our ten beacon councils and new advisory group who will be leading the way in the battle against #Covid19 at a local level. #WhackTheMole £300 million additional funding for local authorities to support new test and trace
(30) Funding charities and helping those most affected by lockdown -BEAT helping YP eating disorders/bereavement/Every Mind Matters. We’ve also been fighting #COVID19
(31) Time to turn tables on #COVID19 If you have symptoms, you will be tested. If + we will trace your recent contacts who will be asked to self isolate for 14days. Local outbreaks will be handled by LAs to prevent a further national lockdown. We’re coming after you, #coronavirus

The use of words such as “fight,” “soldiers,” “enemy,” “beat,” and “battle” in these tweets reflect the metaphor that the nation was in a war against the disease. While this metaphor gives the impression that the nation is united against a common enemy, which might represent strength, the use of war metaphors when referring to health issues has been widely criticized in the literature. For example, Hauser and Schwarz [52] state that war metaphors can have a negative impact on patients and their families.

One of the above tweets uses a seemingly different metaphor; namely, tweet (31), which includes the phrase “turn tables on” COVID-19. While this metaphor is not a war metaphor, it is similar to such metaphors in many ways. First, in both metaphor types the disease is viewed as a strong opponent; one that is formidable and capable of launching an attack. Another similarity between this metaphor and the war metaphor is the personification of the disease. The disease is treated almost like a human being who has power, influence, and is able to sit at a table just like us and, thus, we must turn the table on it. Another, more important similarity between the two metaphors is that they are both conflict-based metaphors. The phrase “turn tables on” originates from gaming discourse which, like war, is a conflict-rich discourse. Strong metaphors such as these conflict-based ones can be suitable for the topic in question. In political discourse, speakers might seek to utilize a range of tactics, including coercion, to achieve their goals [7]; the HOs’ goal was to induce certain reactions from the public, hence the use of conflict-based language.

The Arabic sub-corpus does not contain the same personification of the disease. On the contrary, the disease is perceived as a hurdle; an inconvenience that we must eliminate or surpass. See the following examples:

(32) To every person: take the vaccine; take the step, please.
(33) We’ve spent a year in this pandemic, and this is our plan to get out of it. Take the step and begin your journey; take the vaccine.
(34) I urge everyone to cooperate by following the health precautions. We are all in one boat; negligence from some affects all.
(35) When communicating with others, being able to see your eyes is enough. We thank you for your commitment to wearing the face mask outside your home, because with everyone’s cooperation we will overcome the pandemic, God willing.

In the above tweets, the SHO frames a situation in which all people are on a journey through life together, and the disease is presented as a hurdle that must be cleared, as an obstacle that has narrowed a path but will soon be negotiated, or as a rough sea through which the people must navigate their way. In other words, overcoming the pandemic is represented as a continuation of an original journey; the pandemic has slowed progress, and people must “take a step” to recommence the journey.

The SHO’s tweets contain eight instances of metaphoric reference to the pandemic; they represent 13.5% of his entire corpus. Six of these tweets feature the journey-through-life metaphor discussed above. The remaining two tweets, however, feature a war metaphor. Both of these tweets featured the text translated below.

(36) Staying at home is our strongest weapon–God willing–for overcoming COVID-19.

On further inspection of this tweet, we found that the same text had been used by many Saudi government accounts across various social media networks and on official government websites. The same text was repeated in tweets by the Saudi Minister of Education, Minister of Foreign Affairs, and Minister of Finance. An English version of this tweet is also present on Twitter, meaning the Arabic version may have been a translation of an original English tweet. As this text may not have been composed by the SHO himself, we cannot count these two tweets as part of SHO’s use of metaphor; however, we can say that this metaphor may have arrived in the Arabic corpus through translation. The SHO may have used this metaphor as a strategy to convince people to adhere to regulations imposed to combat the pandemic. Politicians use various measures, such as strong metaphor, direct imperatives, and even coercion, to achieve certain reactions from the public [7]. The introduction of the war metaphor to Arabic might be a manifestation of these measures. Notably, this metaphor has been introduced in Arabic at a time when it appears to be in decline in English, as highlighted by [7].

## Conclusion

This study comprised an investigation of the crisis-response communication strategies applied by two HOs–specifically, how both sought to manage the COVID-19 crisis and communicate to the public the procedures that should be followed during the pandemic to mitigate the effects of this novel health hazard. It is important to compare the strategies of health officials in different countries especially countries that differ culturally and linguistically in order to identify the best procedures for information dissemination during a crisis. A global pandemic required global co-operation and this study provides an in-depth view of how speech acts and metaphor are used in Arabic and how this use compares to how they are used in English. However, the study is limited by the number of tweets each HO chose to write. The SHO did not produce many tweets as the SHO did, so another study may compliment the findings of this one. Also, Investigation of other cultures might reveal different results, which suggests the importance of conducting similar studies based on data from different languages.

We found that both HOs in Saudi Arabia and Britain utilized communication strategies that are suggested by previous research for good crisis information dissemination. For example, Su et al. [53] suggest that health officials need to “develop fact-based, transparent, and accountable messaging.” Both BHO and SHO used their Twitter accounts to reach out to the public. Their accounts served as a method to send messages to the public that are fact-based and accountable. They used persuasive tactics using a variety of speech acts. Also, both HOs communicated to their respective audiences the procedures suggested by the WHO which shows that they followed another strategy suggested by Su et al. [53]: to “leverage international collaboration for consistent messaging and comprehensive crisis communication.” The key word analysis shows that the messages they both delivered were consistent even though they differed in tackling different issues surrounding these messages.

Contrastively, there was some divergence between the HOs communication styles and previously suggested procedures in the literature. For example, Hyland-Wood et al. [47] suggest openness and frankness as the most effective strategy government officials must follow to encourage people to follow certain procedures. However, the strategies followed by the two HOs showed some indirectness. This appears in the lack of declaratory speech acts, the small number of commissive speech acts they both employed, and the prevalence of indirect speech acts, especially in the SHO data.

There were also some further similarities in how they used speech acts; for example, the prevalence of the use of assertions. Elliott-Maksymowicz et al. [19] found assertions predominate in tweets, which accords with our findings. Further, they reported that political arguments on Twitter are often of a very simple nature, featuring assertions in simple sentences; simple language increases the possibility of one’s intended meaning being understood [54]. This explains why assertions appear extensively in our data. However, we did find some differences between the two HOs’ use of speech acts. The BHO used rhetorical questions, while the SHO did not. In political discourse, rhetorical questions are “used to make a point rather than to elicit an answer” [55], and this is exactly how they were used by the BHO. Also, the both HOs used empathy as a strategy for communication. However, they differed in the type of empathetic procedures they utilized. For example, the BHO used expressive speech acts to express her feelings and build solidarity with her audience. On the other hand, the SHO used indirect directive speech acts through the use of declarative sentences and thanking procedures. People-centered and empathetic persuasion is another strategy suggested by Su et al. [53].

The aspect for which we observed the greatest divergence between the two HOs’ tweets was metaphor. In general, we can say that the BHO used conflict-based metaphors; namely, war and gaming metaphors. The SHO, on the other hand, used metaphors that reflected life as a journey that had been interrupted by the pandemic. However, the SHO’s (and other government officials in Saudi Arabia) use of a conflict-based metaphor (weapon) may indicate that, when seeking to implement extreme health measures, such as quarantine, it is necessary to use extreme conflict-based metaphors to convince people. This reminds us of Semino et al.’s work [9, 56], in which they proved that the effectiveness of conflict-based metaphors depends on the context in which they are used. Violence metaphors can be disempowering, but here they serve a purpose of convincing people to follow procedures in the same way that soldiers are expected to follow procedures in wars.

We also found that the discourse the HOs used when referring to the pandemic featured characteristics of both health communication and political discourse. The similarity to health communication relates to the fact that the HOs utilized directive speech acts to tell patients/audiences the procedures they should follow to achieve the desired conclusion: healing of patients and ending of the pandemic. On the other hand, this discourse is similar to political discourse in that it utilized rhetorical questions and assertions to convince people to perform certain behaviors favored by the officials. A similarity between health communication and political discourse in English is the use of war metaphors. Politicians tend to frequently use war metaphors [57, 58], and such metaphors are also common in health-care discourse [10]. While war metaphors were not prevalent in the Arabic corpus, we saw that such metaphors might be arriving in Arabic through translation, as has happened previously in Malaysia and Singapore [59].

The tweet data included a number of images such as photographs, info graphs, emojis, and maps. Although we did not include these images in the present paper except to help in understanding the intended meaning of some tweets, images can be used to enforce and complement speech acts. For example, in the BHO’s assertion that her mother had received the vaccine, she strongly verified this by including with the tweet a photo of her mother. How speech acts are reinforced by imagery is a subject for further study. Does the type of speech act correlate with the type of imagery used? For example, are assertions accompanied by info graphs and thanks by emojis of hearts and flowers? Are assertions not enough to persuade people and we need to show them photographic and video evidence so that they believe what we say to them even if the information is from a reliable source like a health official?

This study identifies different strategies used by HOs, and shows that these strategies are compatible with the ones suggested by previous research. However, it would be beneficial to examine the impact of these different strategies on the public using experimental studies that expose participants to different conditions and measure the effect of those communication strategies (See [60]). Other questions could be explored: Was the directness of the BHO more efficient than the indirectness of the SHO? Or vice versa? We leave this for future investigation. Addressing emergencies to the public, particularly for a crisis such as COVID-19, needs leaders to adopt the risk communication approach and develop a consensual narrative that does not rely on a coercion strategy. A narrative of this nature should include factual and open information about uncertainty. During the COVID-19 crisis, those leaders who were able to gain public trust were those who advocated participatory public communication and fact-checked information.

## Supporting information

S1 File English data: The tweets of the British health official analyzed in this paper. (DOCX)

S2 File Arabic data: The tweets of the Saudi health official analyzed in this paper. (TXT)
