# Correction: Crises information dissemination through social media in the UK and Saudi Arabia: A linguistic perspective

**DOI:** 10.1371/journal.pone.0296602

**Published:** 2024-01-02

**Authors:** The PLOS One Staff

There are errors in the Funding section. The correct Funding statement is: The researchers would like to acknowledge Deanship of Scientific Research, Taif University for funding this work. The funders had no role in study design, data collection and analysis, decision to publish, or preparation of the manuscript.

The publisher apologizes for the error.]
