# Supplementary material for: Crises information dissemination through social media in the UK and Saudi Arabia: A linguistic perspective
Source: PLoS One. 2023 May 5;18(5):e0284857. doi: 10.1371/journal.pone.0284857 (PMC10162563; doi:10.1371/journal.pone.0284857)
Supplement: S1 File — (DOCX) [file pone.0284857.s001.docx]

Boris Johnson: The Oxford vaccine shows why we and the world need Britain to be global

We’ve just made radical changes to what happens to perpetrators of domestic violence in the home, a National Strategy takes this onto the streets and of course, we want women’s voices to be a part of that. The absolute commitment to end [#VAWG](https://twitter.com/hashtag/VAWG?src=hashtag_click) already at the heart of gov policy

3 Those who cite low daily diagnosed [#COVID19](https://twitter.com/hashtag/COVID19?src=hashtag_click) cases as reason to exit lockdown now, miss the point. They are low, because of lockdown. Those who cite all cause daily death rates being lower than normal, ditto. [#HandsFaceSpace](https://twitter.com/hashtag/HandsFaceSpace?src=hashtag_click) protects us from many viral and bacterial infections

All of my life I’ve walked the dogs with the strap of the leads in my hand and the buckles swinging, in case I need to defend myself. I realised in this incredibly sad week, that’s not normal and every other female dog walker does the same thing. Things have got to change.

I’ve switched comments off on my feed and it will stay that way. I wouldn’t invite 92k people into my garden to scream abuse at me, so I won’t be having it on my phone either. It’s the reason so many female MPs won’t touch Twitter. I think this is a good compromise.

One year ago. Five people had died. I remember the surreal moment of panic when a senior PHE clinician called to tell me I’d tested positive. “I’d have sworn you would test negative” at exactly the same moment I realised I had given it to my poorly 84yo mum. By

[@jessicaelgot](https://twitter.com/jessicaelgot)

In today’s ⁦

[@Daily_Express](https://twitter.com/Daily_Express)

⁩ We must continue to expand mental health services for Generation Lockdown - Nadine Dorries | Express Comment | Comment | [http://Express.co.uk](https://t.co/zuA9VWJNsz?amp=1)

<https://www.express.co.uk/comment/expresscomment/1406533/generation-lockdown-mental-health-services-nadine-dorries>

Countries with high levels of obesity have the highest rates of [#Covid](https://twitter.com/hashtag/Covid?src=hashtag_click) death.

Thank you though for at least highlighting that PPE was an issue every county had to deal with at the start of a global pandemic and that it wasn’t just the U.K. We did amazingly well to have dealt with the problem in this country as efficiently and as fast as we did

That’s me done (syringe emoji)


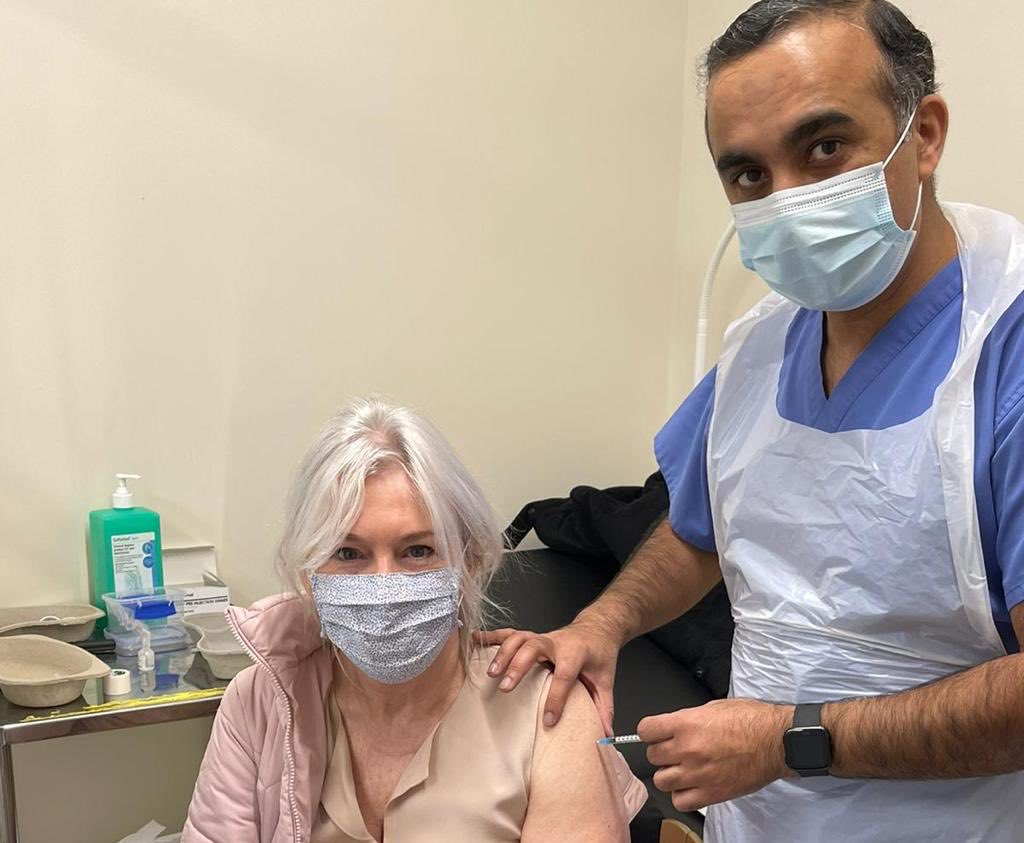


This photograph is from a Spanish hospital.

(photo deleted)

Those calling for lockdown to be lifted as soon as we reach a % of adults vaccinated should heed this cautionary reality check from

[@MattHancock](https://twitter.com/MattHancock)

Absolutely no one in Government wants us to be in lockdown for a day longer than we have to be.

It could be up to [#NHS](https://twitter.com/hashtag/NHS?src=hashtag_click) nurses and doctors working long hours in full PPE though if you infect one of them with [#Covid](https://twitter.com/hashtag/Covid?src=hashtag_click) (woman facepalming emoji)

Thank you Fred!

(quoted tweet)

Well done Prime Minister @BorisJohnson @Conservatives @NadineDorries and team in keeping to aim of 15 million vaccinations by today! Kept promise as usual.

You were given votes. All but 13 MPs supported the Gov measures.

This has been a challenging time for our children & young people and it’s understandable if they’re feeling anxious or low. Get your children to express themselves this & reach out for support & resources: [#Childrensmentalhealthweek](https://twitter.com/hashtag/Childrensmentalhealthweek?src=hashtag_click) [https://drive.google.com/file/d/1_ffkrNVj33-W-Z26y0ckv_BtaAxZ0-rE/view](https://t.co/0bVWQX7rzR?amp=1)

Not sure if Robert was trying to say that there was a causal link between murder/knife crime and mental health here? The two groups affected by pandemic are young women aged 15-26 especially with eating disorders and those with pre existing MH illness

https://twitter.com/NadineDorries/status/1357267292652994560?s=20

I have to respond to the facts. We have invested £2.3b into MH services inc an additional £500m in SR to accelerate introduction of MH task forces in schools and services focused on the 2 main groups affected by pandemic.

https://twitter.com/NadineDorries/status/1357266019870519296?s=20

A refreshing change from ⁦

[@BBCNews](https://twitter.com/BBCNews)

⁩ This excellent round up is actual news/fact not pontification/opinion. Europe's Disaster Week | BBC Outside Source - YouTube

https://twitter.com/NadineDorries/status/1356749238810591232?s=20

{vaccine deals}

https://twitter.com/NadineDorries/status/1356749238810591232?s=20

Have campaigned on this issue since 1st day I became MoS in

[@DHSCgovuk](https://twitter.com/DHSCgovuk)

in firm belief that women’s voices in the healthcare setting are not always well heard and women not listened to. Trials halted due to [#Covid](https://twitter.com/hashtag/Covid?src=hashtag_click) must now recommence with aim of universal testing at 35-37w the aim

23 Last week I wrote to all trust CEOs asking them to follow the 2017 RCOG guidelines as closely as possible using ECM (enriched culture medium) testing in order to provide the highest standard of care possible for both mother and baby. [#Patientsafety](https://twitter.com/hashtag/Patientsafety?src=hashtag_click)

24 He’s the (star emoji) of Mid Bedfordshire and our prayers go out to him and all those in hospital with [#Covid](https://twitter.com/hashtag/Covid?src=hashtag_click) this weekend. (@CaptainTomMoore)

25 As

[@SophyRidgeSky](https://twitter.com/SophyRidgeSky)

has just reminded us, last year, Labour campaigned for us to join the EU vaccines programme. Not only was that the wrong call, the fundamental values which underpinned their campaigning and decision making do nor deliver the best outcomes for the British people.

26 .

[@UKLabour](https://twitter.com/UKLabour)

are calling for the government to override the recommendations from the Joint Committee on Vaccinations and Immunisations. They apparently know better. (woman facepalming emoji)

27 The last few months have been tough and it’s important we are all looking after our mental health and wellbeing – Every Mind Matters resources offer tailored advice, practical tips on sleep and self-care.


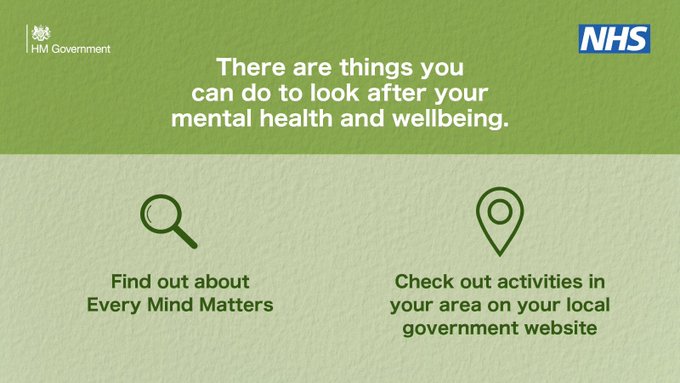


28 Grieving has been so difficult for those who have lost loved ones, some not even able to say goodbye. We've given over £10.2m to charities, including bereavement charities, since March to help + ensure services have been there for those who need them.

29 I understand the necessary restrictions in place over the last year have made grieving difficult for those who have lost loved ones. We've given over £10.2m to charities, including bereavement charities, since March to help ensure services have been there for those who need them.

30 As lockdown continues, many of us will be feeling fed up and anxious - this is understandable. I urge anyone who’s struggling to come forward for help – these services are here to support you

The

[@DailyMirror](https://twitter.com/DailyMirror)

group of newspapers engaged in some highly political and dangerously misleading fear mongering over the past few days. Good to see this )downpointing triangle emoji)

https://twitter.com/NadineDorries/status/1353756006652776449?s=20

32 World leading vaccine rollout. [#NHS](https://twitter.com/hashtag/NHS?src=hashtag_click)


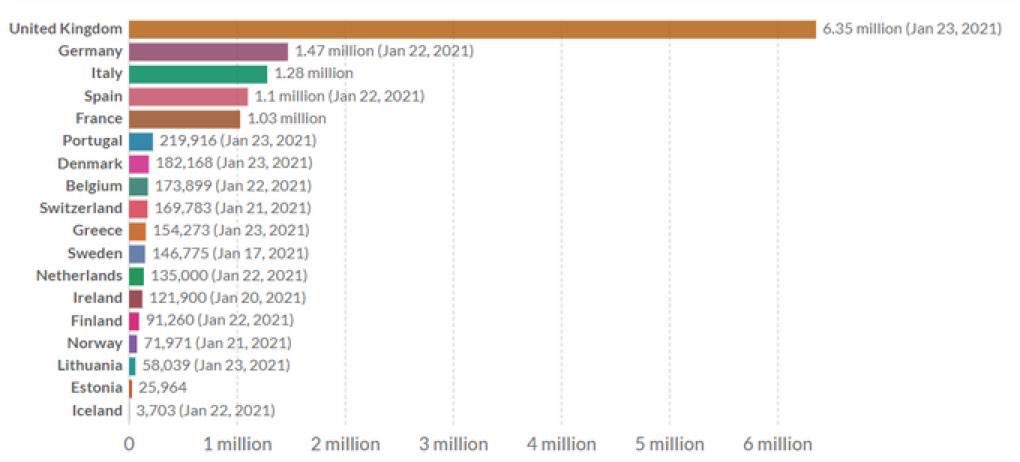


33 And this

(downpointing triangle emoji)

from

[@Steven_Swinford](https://twitter.com/Steven_Swinford)

[@thetimes](https://twitter.com/thetimes)

Essential to have [#Covid](https://twitter.com/hashtag/Covid?src=hashtag_click) [#Truthseekers](https://twitter.com/hashtag/Truthseekers?src=hashtag_click) on here.

https://twitter.com/NadineDorries/status/1353624287274541061?s=20

.

[@piersmorgan](https://twitter.com/piersmorgan)

34 totally unfair. Our T+T was set up to service 66m people for a new virus from scratch and now 85% receive results within 24hrs. Well done

[@theresecoffey](https://twitter.com/theresecoffey)

on

[@GMB](https://twitter.com/GMB)

35 Almost half a million on one day. (British flag emoji) + (syringe emoji)


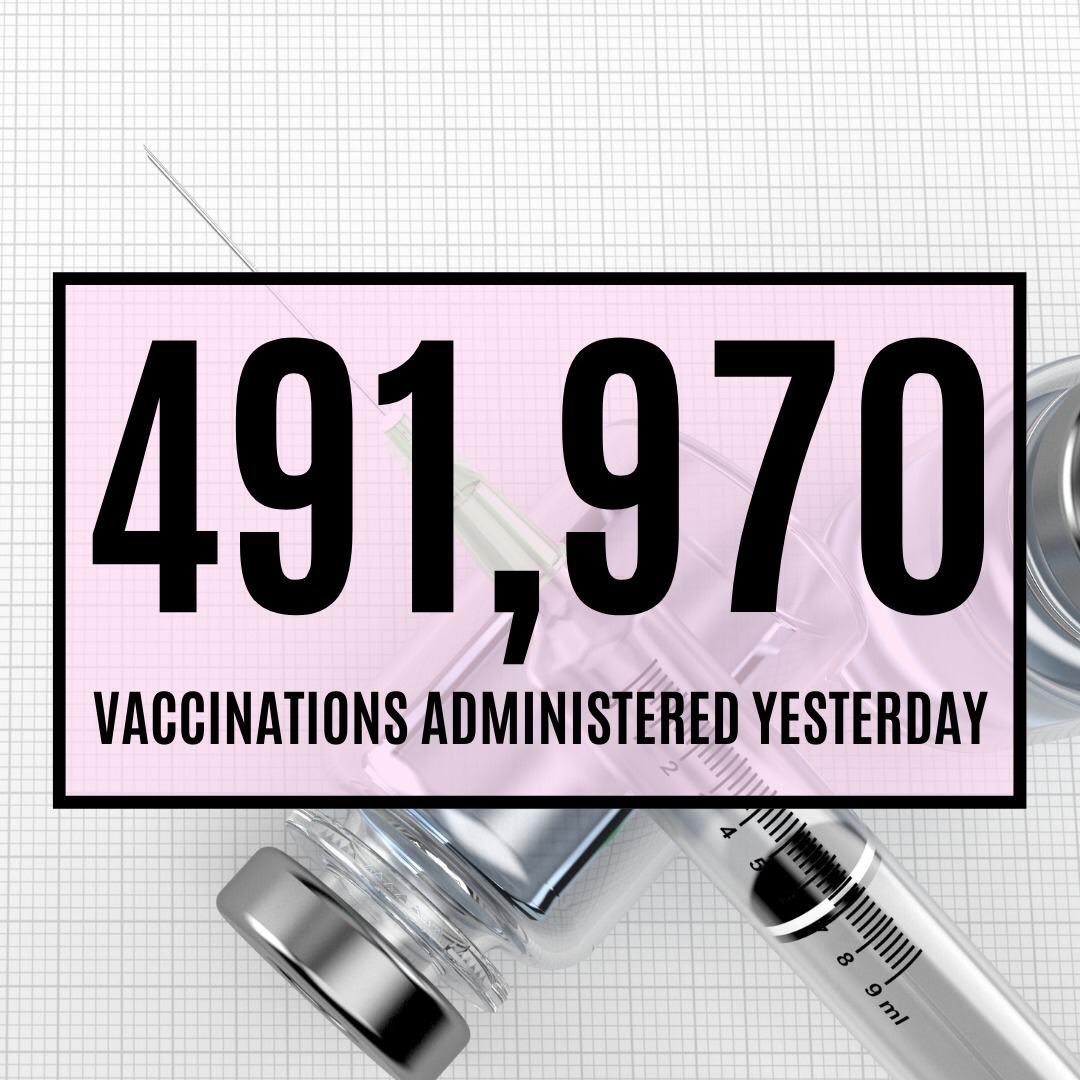


https://twitter.com/NadineDorries/status/1353407001980776453?s=20

36 World leaders in [#Covid](https://twitter.com/hashtag/Covid?src=hashtag_click) vaccine rollout.


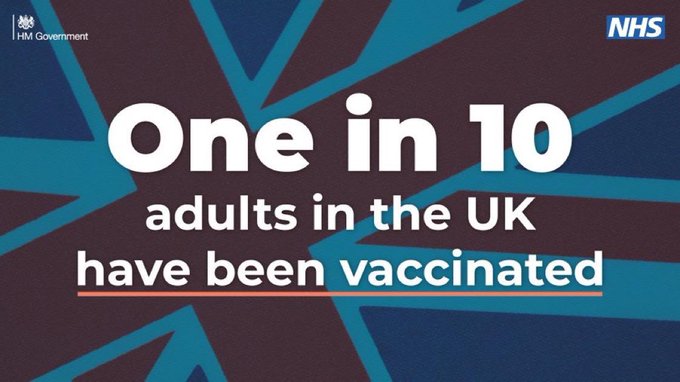


<https://twitter.com/NadineDorries/status/1352679927297024000?s=20>

37 8 reasons the UK leads Europe’s coronavirus vaccination race – POLITICO

https://twitter.com/NadineDorries/status/1349988406886076417?s=20

38 We are miles ahead (syringe emoji)


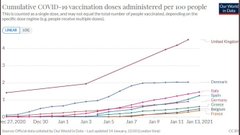


https://twitter.com/NadineDorries/status/1349753842951458823?s=20

39 This is totally untrue. As a well known

[@UKLabour](https://twitter.com/UKLabour)

activist, you still have a responsibility to be accurate with the facts.

Quote Tweet

https://twitter.com/NadineDorries/status/1349707654009118721?s=20

40 Over 3million vaccines into arms across the U.K.


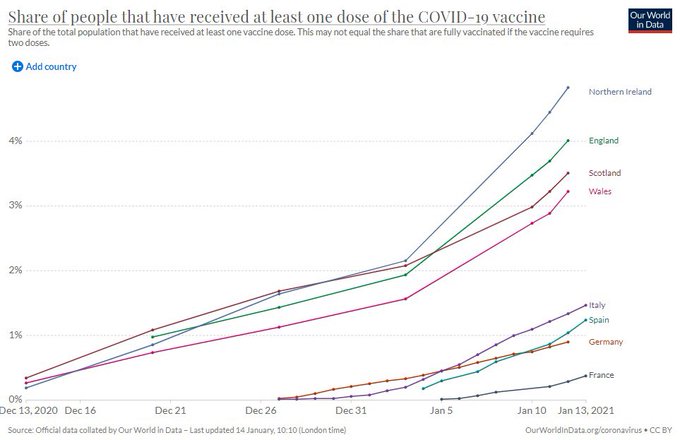


https://twitter.com/NadineDorries/status/1349702323132624897?s=20

41 126 NHS trusts and 44 local maternity units will benefit The training will aim to apply lessons learned both from the pandemic and maternity safety inquiries, including the Ockenden Review – and help overcome disconnect between ‘ward and board’

https://twitter.com/NadineDorries/status/1348998914687561730?s=20

42 A very useful graphic. [#COVIDVaccination](https://twitter.com/hashtag/COVIDVaccination?src=hashtag_click)


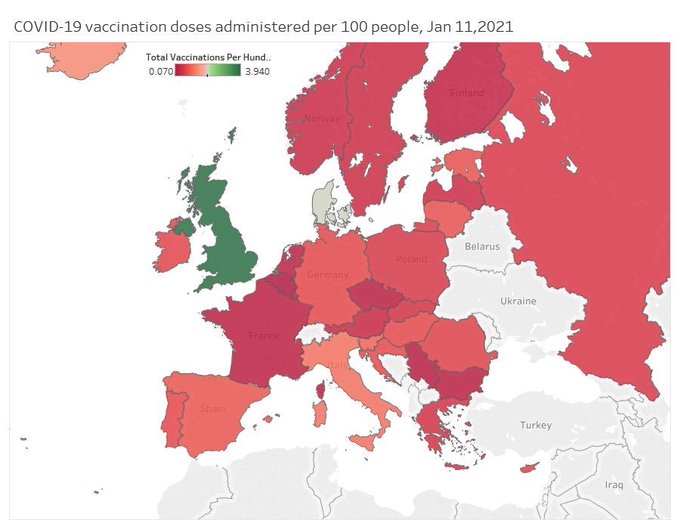


https://twitter.com/NadineDorries/status/1348994111542616064?s=20

43 I have only ever said ‘without a vaccine or treatment,’ there is no such thing as herd immunity. The vaccine is the game changer, but we are no where near that point yet. [#StayHome](https://twitter.com/hashtag/StayHome?src=hashtag_click)

[
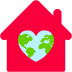
](https://twitter.com/hashtag/StayHome?src=hashtag_click)

https://twitter.com/NadineDorries/status/1348546759576973318?s=20

44 10 million doses, ordered (whit heavy check marker emoji)

https://twitter.com/NadineDorries/status/1347562832792088576?s=20

45 It’s work in progress and we are already at 110,000. Well past your 50k! Thanks for your help, disappointing you have you tag

[@TheLabourParty_](https://twitter.com/TheLabourParty_)

name onto the [#CovidVaccine](https://twitter.com/hashtag/CovidVaccine?src=hashtag_click) and attempt to politicise a great national effort though. [#ShameOnYou](https://twitter.com/hashtag/ShameOnYou?src=hashtag_click)


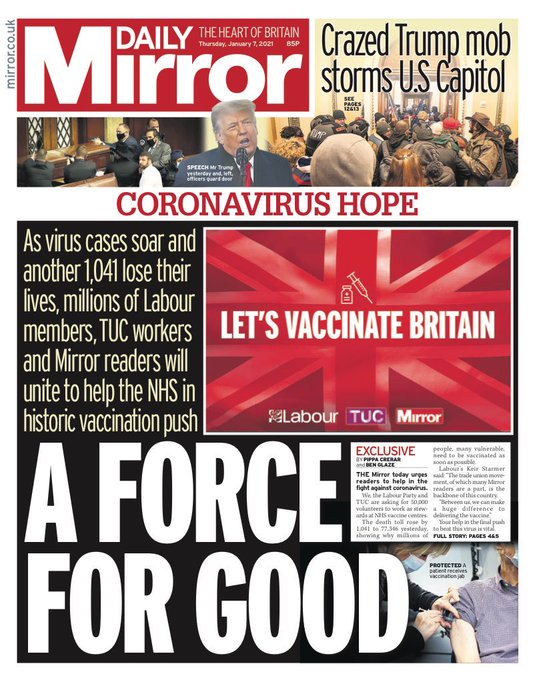


<https://twitter.com/NadineDorries/status/1347096628323348481?s=20>

46 My 84yo mum couldn’t have been happier to be called up for her [#CovidVaccine](https://twitter.com/hashtag/CovidVaccine?src=hashtag_click) today. We have a robust vaccine rollout plan. The first in the world to administer both leading vaccines- the fastest in Europe, and are accelerating fast!


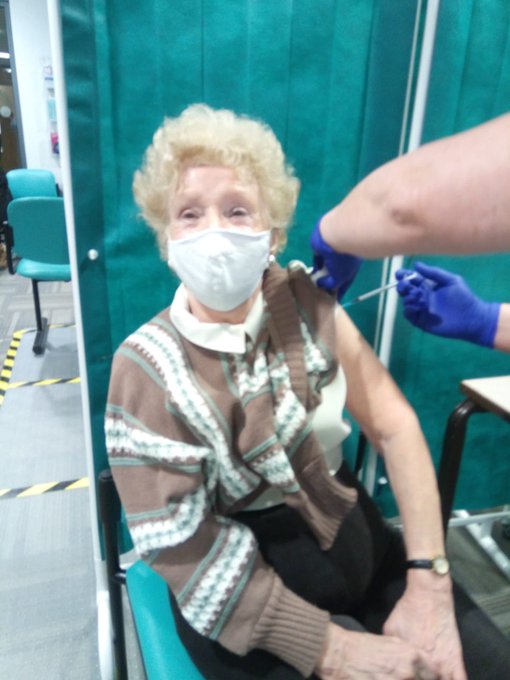


https://twitter.com/NadineDorries/status/1346926227106492423?s=20

47 Hi Kathy, it’s actually a pandemic caused by a mutating killer virus - very different to communism I believe. It’s also about keeping people alive and safe. Thankfully, the vast majority of the population do not share your opinion and understand the reasons behind [#lockdown](https://twitter.com/hashtag/lockdown?src=hashtag_click)

https://twitter.com/NadineDorries/status/1346456671372120065?s=20

48 From JVT ‘If a family has two elderly grandparents and there are two vaccines available, it is better to give both 89 per cent protection than to give one 95 per cent protection with two quick doses, and the other grandparent no protection at all.’

From JVT 49

‘If a family has two elderly grandparents and there are two vaccines available, it is better to give both 89 per cent protection than to give one 95 per cent protection with two quick doses, and the other grandparent no protection at all.’

https://twitter.com/NadineDorries/status/1345665655274688516?s=20

50 Joint Committee on Vaccination & Immunisation finds Pfizer vaccine 89% effective from 15 - 21 days after first dose. Rises to 95% after second dose

51 We’ve updated maternity guidance, balancing controlling COVID-19 infection and allowing women to have one person with them at all appts at all stages. This is an important step for the emotional wellbeing, safety & personalised care for women & their loved ones across the country

52 Be proud. (British flag emoji)


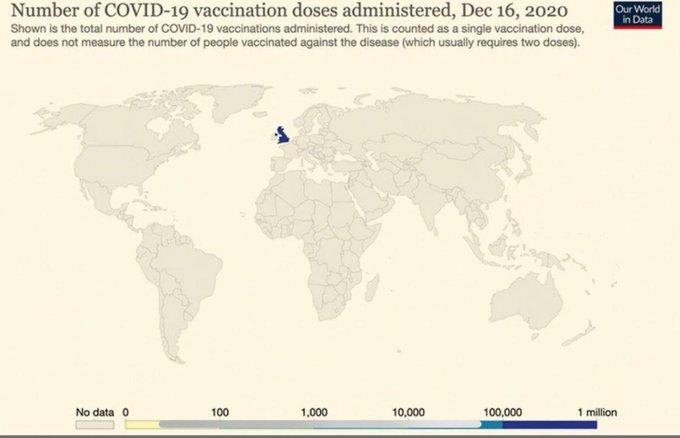


https://twitter.com/NadineDorries/status/1339479408588021760?s=20

53 British scientists have led the world in [#Covid](https://twitter.com/hashtag/Covid?src=hashtag_click) response. In discovering that Dexamethasone reduces Covid mortality, here in the (British flag emoji)

54 we have globally saved in excess of at least a million lives.

https://twitter.com/NadineDorries/status/1336624924790820865?s=20

55 Today has been an emotional day. On our little island, our [#NHS](https://twitter.com/hashtag/NHS?src=hashtag_click) administered the first [#Covid](https://twitter.com/hashtag/Covid?src=hashtag_click) vaccine in the world. I’ve been so proud to be a Minister in

[@DHSCgovuk](https://twitter.com/DHSCgovuk)

during the hardest ten months but today, was a very good day. Prouder of our healthcare workers and all involved

https://twitter.com/NadineDorries/status/1336419340640456706?s=20

56 Just the best news.

https://twitter.com/NadineDorries/status/1335712634021289985?s=20

[57 @SkyNews](https://twitter.com/SkyNews)

pedalling the Fauci comments from yesterday. They are behind with the news. He has since apologised and said “I have great faith in UK regulators”

https://twitter.com/NadineDorries/status/1334758241788710914?s=20

58 No one should be in any doubt about how it is that we can start rolling out the vaccine next week. A month ago, we changed the regulations to exempt us from requiring EU approval. We would still be waiting if we hadn’t. Thanks to [#Brexit](https://twitter.com/hashtag/Brexit?src=hashtag_click) we can now move ahead swiftly and safely

https://twitter.com/NadineDorries/status/1334122869194940419?s=20

59 Remember this?


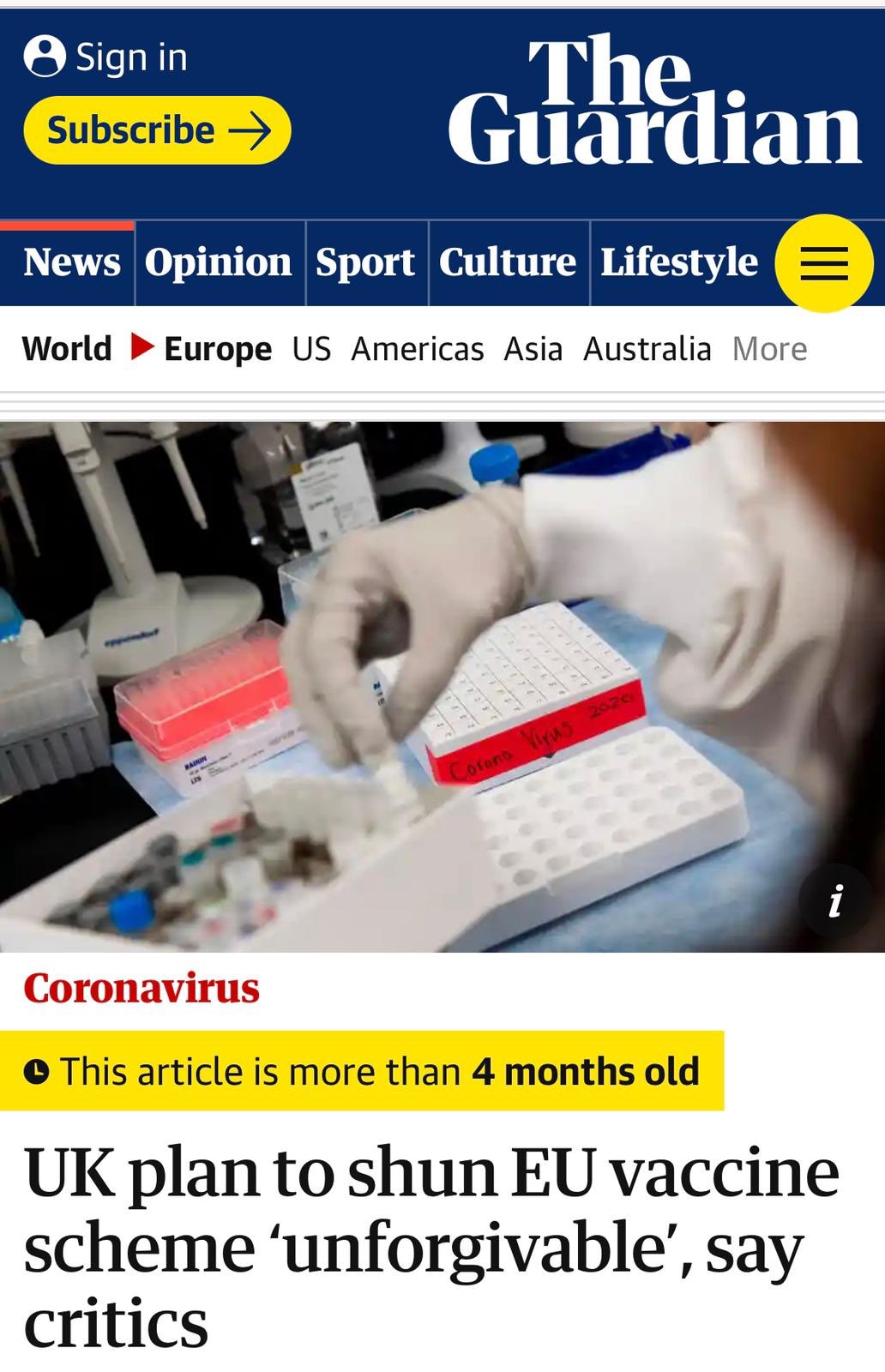


https://twitter.com/NadineDorries/status/1334071349162233857?s=20

60 The news we have all been waiting for with baited breath. The MHRA has formally approved the Pfizer/BioNTech vaccine

(syringe emoji)

61 The U.K. is the first country in the world to have a vaccine supply primed and ready to begin. The NHS will begin vaccinating next week. Rejoice!

https://twitter.com/NadineDorries/status/1334041213842886656?s=20

62 The doubtful case for an impossible Covid-19 cost-benefit analysis  | The Institute for Government

https://twitter.com/NadineDorries/status/1333532970095964161?s=20


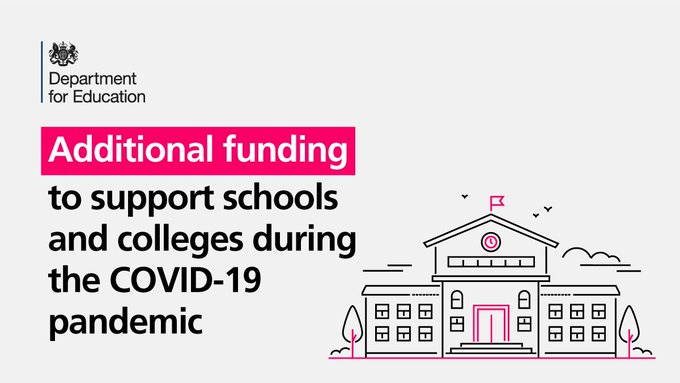


https://twitter.com/NadineDorries/status/1333418583431507972?s=20

63 In today’s MoS from

[@BorisJohnson](https://twitter.com/BorisJohnson)


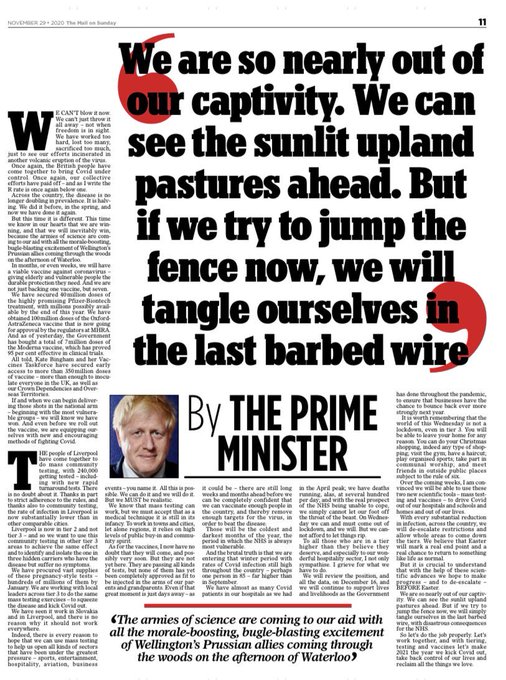


64 Thrilled and delighted at the appointment of

[@nadhimzahawi](https://twitter.com/nadhimzahawi)

to lead the vaccine rollout. Ops experience combined with huge intellect. He’s the right man in the right place. The biggest possible welcome to the team, Nadhim. Let’s get this job done

(syringe emoji) + (collision symbol)

<https://twitter.com/NadineDorries/status/1332678843942002691?s=20>

65 This amazing new blood test announced today, which can detect up to fifty types of cancer in early stages is a game changer. Our [#NHS](https://twitter.com/hashtag/NHS?src=hashtag_click) our

(British flag emoji)

Pioneering and leading from the front. Not just with the [#Covid](https://twitter.com/hashtag/Covid?src=hashtag_click) vaccine, but in all areas of innovation [#AI](https://twitter.com/hashtag/AI?src=hashtag_click) and life saving technology.

Quote Tweet

https://twitter.com/NadineDorries/status/1332240233266601985?s=20

66 Helpful data available on Gov.U.K. The infection rate in the number of over 60s tells us what the demand on hospital beds will be in 14 days time.


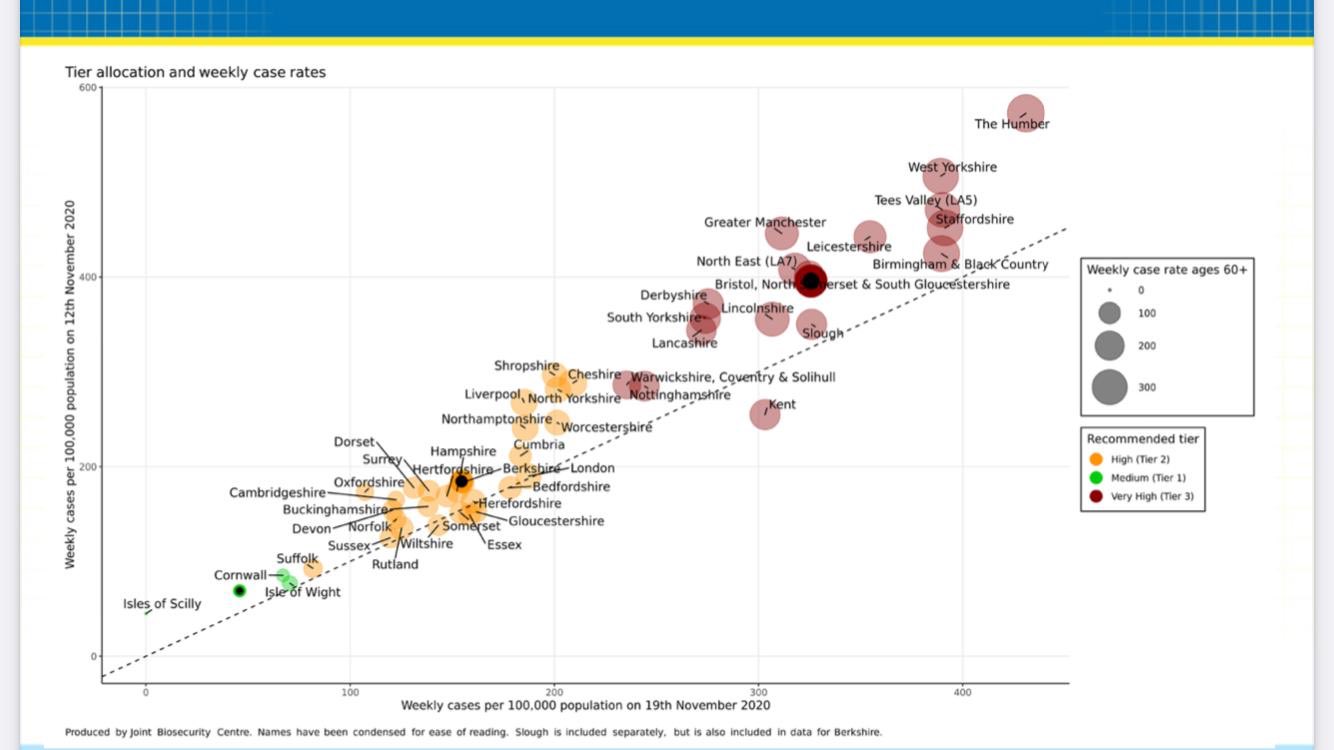


https://twitter.com/NadineDorries/status/1332031908033798148?s=20

67 This government has invested more money into mental health than any government for many decades. £2.3 billion into a long term plan, many millions during Covid and now this (downward arrow)

https://twitter.com/NadineDorries/status/1330485014354669570?s=20

68 This government has invested more money into mental health than any government for many decades. £2.3 billion into a long term plan, many millions during Covid and now this

(downward arrow emoji)

https://twitter.com/NadineDorries/status/1330485014354669570?s=20

69 [We have (british flag emoji)](https://t.co/cmUU5IlnEh?amp=1)


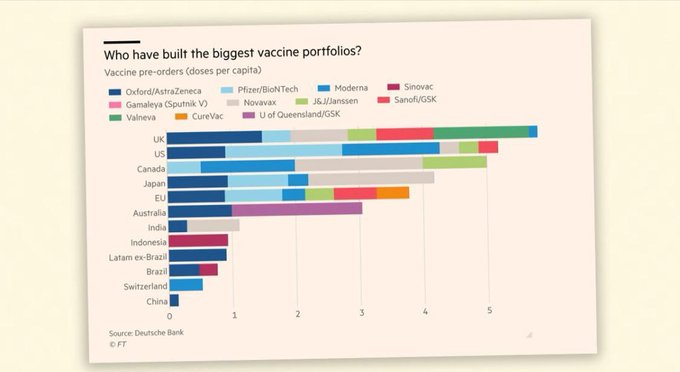


https://twitter.com/NadineDorries/status/1328802676352708611?s=20

70 No doubt that there is a direct correlation between obesity and a high mortality Covid rate. We are one of the most obese nations in Europe. Eat less move more.

https://twitter.com/NadineDorries/status/1328008580105170944?s=20

71 We’re testing more people per head of population than any other country. A key (boxing hand emoji)

72 in fight against [#COVID19](https://twitter.com/hashtag/COVID19?src=hashtag_click) Remember when we were told that bcse we had left the EU we would be at the back of the queue for vaccines? We were at the front and have secured 40m of the Pfizer vac (syringe emoji)

https://twitter.com/NadineDorries/status/1327178691051483137?s=20

73 One person to single out for praise when we receive the Pfizer vaccines, Kate Bingham, chair of the vaccines task force. As Professor Sir John Bell, Regius professor of medicine at Oxford university, said the UK had “only got 30m doses of the Pfizer/BioNTech because of her”.

https://twitter.com/NadineDorries/status/1326525541176979458?s=20

74 It’s actually now 40m doses. As a leader in the world of life sciences for 30 years, she has worked around the clock since May, for free, to make this happen.

https://twitter.com/NadineDorries/status/1326525844236464129?s=20

75 The funding wasn’t for PR, let alone personal PR but to encourage and educate members of the public to sign up to the Covid19 vaccine trials and it was a huge success with 30,000 registered

<https://twitter.com/NadineDorries/status/1326526820469133313?s=20>

76 Just walked past a TV screen and saw a Dr from Liverpool arguing against mass testing. We’ll leave that one to the many 1000s of Drs who have been asking for it.

(woman facepalming emoji)

https://twitter.com/NadineDorries/status/1325845578631303169?s=20

77 Every life lost to suicide, is a tragedy, however this data showing zero increase in suicide figures since lockdown should be reassuring to those who have been concerned regarding the mental health impact of [#coronavirus](https://twitter.com/hashtag/coronavirus?src=hashtag_click) and [#lockdown](https://twitter.com/hashtag/lockdown?src=hashtag_click)

https://twitter.com/NadineDorries/status/1325802615754678274?s=20

78 We will continue to monitor the data and evaluate services, including the introduction of a wellbeing and mental health winter plan which I shall be announcing this week. I’m the meantime.

https://twitter.com/NadineDorries/status/1325804002655805440?s=20

79 It is important people keep reaching out for support if they need it. If you’re in crisis, round the clock support is available in your local area. Visit the NHS website to find services near you or visit Every Mind Matters web site

https://twitter.com/NadineDorries/status/1325804178061496326?s=20

1/4 I want to see Moira Garwood take that bow! People of Liverpool, you have been amazing. Leading the way in mass testing, you hold the key to unlocking the rest of the country and you are generously stepping up to play your part for all of us.

https://twitter.com/NadineDorries/status/1325041584912658434?s=20

81 My mother in law told me that during WWII, in Bootle, families and the home guard flocked to the shore to build wooden guns and pointed them to the sky to fool the enemy flying overhead. Robert Hardman told me he could sense that spirit amongst the people queuing to be tested

https://twitter.com/NadineDorries/status/1325041888227954688?s=20

82 3/4 2000 of our armed forces, from twenty locations across the U.K. arrived in Liverpool with two days notice. They have demonstrated as they always do, how and why it is we have the best armed forces in the world.

https://twitter.com/NadineDorries/status/1325042199323611138?s=20

83 4/4 Being a scouse girl, I love Moira’s comments- of course scouse girls are going to turn out to be tested by a good looking soldier! They know how to have a laugh, that’s how they got through the blitz. in the cold long queue, scousers were resolute and they make me proud.

https://twitter.com/NadineDorries/status/1325042729596936192?s=20

84 The man with the biggest standing in Liverpool since Bill Shankly, Jurgen Klopp, leads the call for the people in Liverpool to get the city tested.

<https://twitter.com/NadineDorries/status/1324993092731228163?s=20>

85 Just to be clear, dog groomers can remain open if [#Covid](https://twitter.com/hashtag/Covid?src=hashtag_click) secure. They are not on the list of businesses to close. Dog grooming prevents matted (painful) coats and long nails. Remaining open is an ani@al health and welfare issue. (dog emoji)

https://twitter.com/NadineDorries/status/1324770868069892096?s=20

86 It’s understandable that social restrictions may make you feel anxious, but there are simple steps we can all take to look after our mental wellbeing. It’s down to us to create our own personalised support plan. We know it works so be prepared head to –

<https://twitter.com/NadineDorries/status/1324385459800330240?s=20>


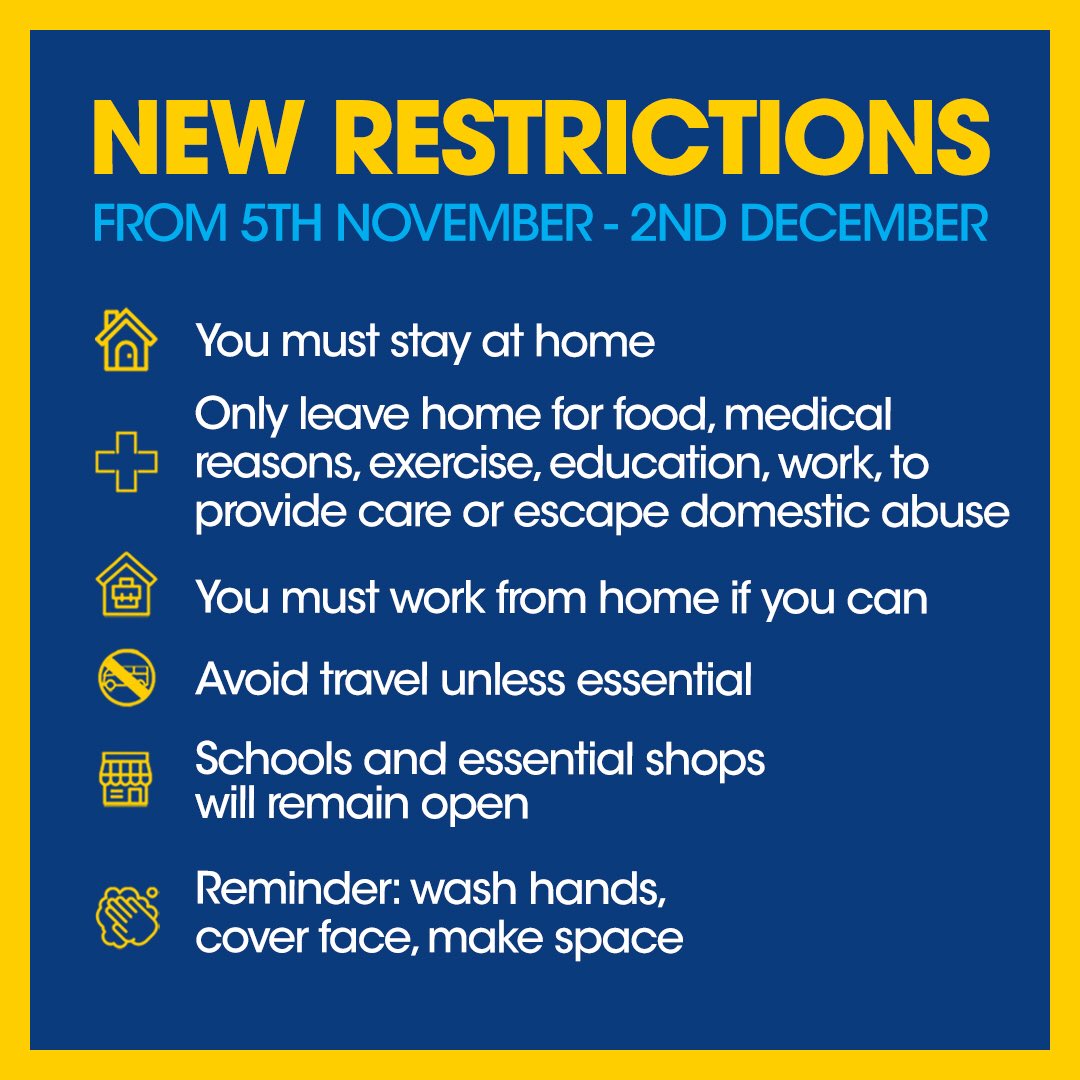


https://twitter.com/NadineDorries/status/1324291204788486147?s=20

87 1/2 I am really happy to let all mums know that from Thursday 5 November children under school age will be exempt from the 15-person limit on support groups. This change will ensure that up to 15 parents can attend a support group with their babies.

https://twitter.com/NadineDorries/status/1323659505838817282?s=20

88 2/2 And that the valuable parent-baby support provided by these groups is not disrupted during the November restrictions.

https://twitter.com/NadineDorries/status/1323659606221115394?s=20

And dads too! Sorry dads

https://twitter.com/NadineDorries/status/1323659877458300928?s=20

89 Liverpool in October right now and without lockdown, heading to a hospital near you. "People are right to say that these are pressures that occur every winter, but this time it's just a lot worse. This is winter plus, plus, plus," says Dr Zuzan.

Quote Tweet

https://twitter.com/NadineDorries/status/1323557068641603589?s=20

90 My home city of Liverpool is about to run the first pilot for lateral flow [#Covid](https://twitter.com/hashtag/Covid?src=hashtag_click) testing for anyone, symptoms or not- results in 15mins. Huge for schools, care homes, hospitals etc. Army now setting up 30 fixed and mobile testing units across the city. False + only 1:1000

https://twitter.com/NadineDorries/status/1323541425187966978?s=20

91 We learn more about this virus every day. It attacks the central nervous system and affects the mental health of young people from long [#Covid](https://twitter.com/hashtag/Covid?src=hashtag_click) - bereavement, PTSD from being ventilated. This is why lockdown is necessary - there are MH consequences from allowing Covid to run free.

https://twitter.com/NadineDorries/status/1323394040784654336?s=20

92 Mental health of coronavirus sufferers is being ignored, Royal College of Psychiatrists warns | The Independent

https://twitter.com/NadineDorries/status/1323323393131876352?s=20

93 Sarah-Jane, you have been beyond amazing. An inspirational role model to all young women and men who have watched you work and achieve. Your return to

[@BWC_NHS](https://twitter.com/BWC_NHS)

is our loss. Good luck in all that you do.

Quote Tweet

https://twitter.com/NadineDorries/status/1323308096861720580?s=20

94 New prediction of daily new infections, especially in over 60s and demand on hospital beds is far in excess of prior reasonable worst case scenario.

https://twitter.com/NadineDorries/status/1323031277218471937?s=20

95 A really excellent explanation with visuals demonstrating how [#COVID19](https://twitter.com/hashtag/COVID19?src=hashtag_click) transmits and infects. Aerosol transmission of Covid-19: A room, a bar and a classroom: how the coronavirus is spread through the air | Society | EL PAÍS in English

https://twitter.com/NadineDorries/status/1322998184545800199?s=20

96 Children under school age who are with their parents will not count towards the limit on two people meeting outside. This will mean that a parent can see a friend or family member with their baby or young children.

https://twitter.com/NadineDorries/status/1322909509355003910?s=20

97 Children and adults who are dependent on round-the-clock care, such as those with severe disabilities, will also be included. The guidance will be updated to reflect this.

https://twitter.com/NadineDorries/status/1322909672525897730?s=20

98 See? If only we had a crystal ball and could actually see how many over 60s would be infected, the positivity rate, the infection rate and the subsequent lag giving us the 14day anticipated demand on hospital beds on any particular day, three weeks in advance.

Quote Tweet

https://twitter.com/NadineDorries/status/1322490387978723329?s=20

99 In today’s ⁦

[@thetimes](https://twitter.com/thetimes)


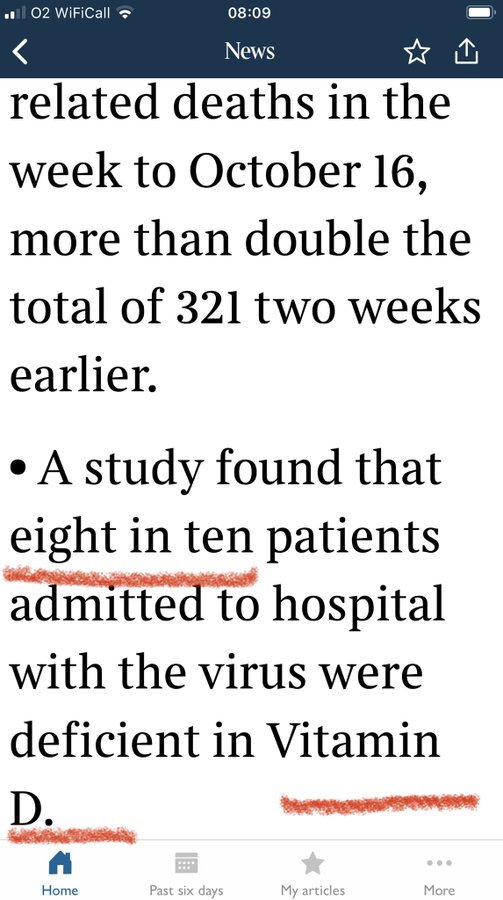


https://twitter.com/NadineDorries/status/1321364414373744642?s=20

100 Hey John, we scousers like fair play. Here’s a link to the money councils were given to help disadvantaged families with food vouchers, etc long before the vote stunt kicked in. This does not include the extra £1b support Liverpool listed [https://gov.uk/government/publications/coronavirus-covid-19-local-authority-emergency-assistance-grant-for-food-and-essential-supplies/coronavirus-covid-19-local-authority-emergency-assistance-grant-for-food-and-essential-supplies…](https://t.co/ho5VElhFmR?amp=1)

Quote Tweet

https://twitter.com/NadineDorries/status/1319996634105847809?s=20

101 Since the beginning of the [#COVID19](https://twitter.com/hashtag/COVID19?src=hashtag_click) crisis

[@letstalkcentral](https://twitter.com/letstalkcentral)

has received an extra £24.25m

https://twitter.com/NadineDorries/status/1319957105676308481?s=20

[
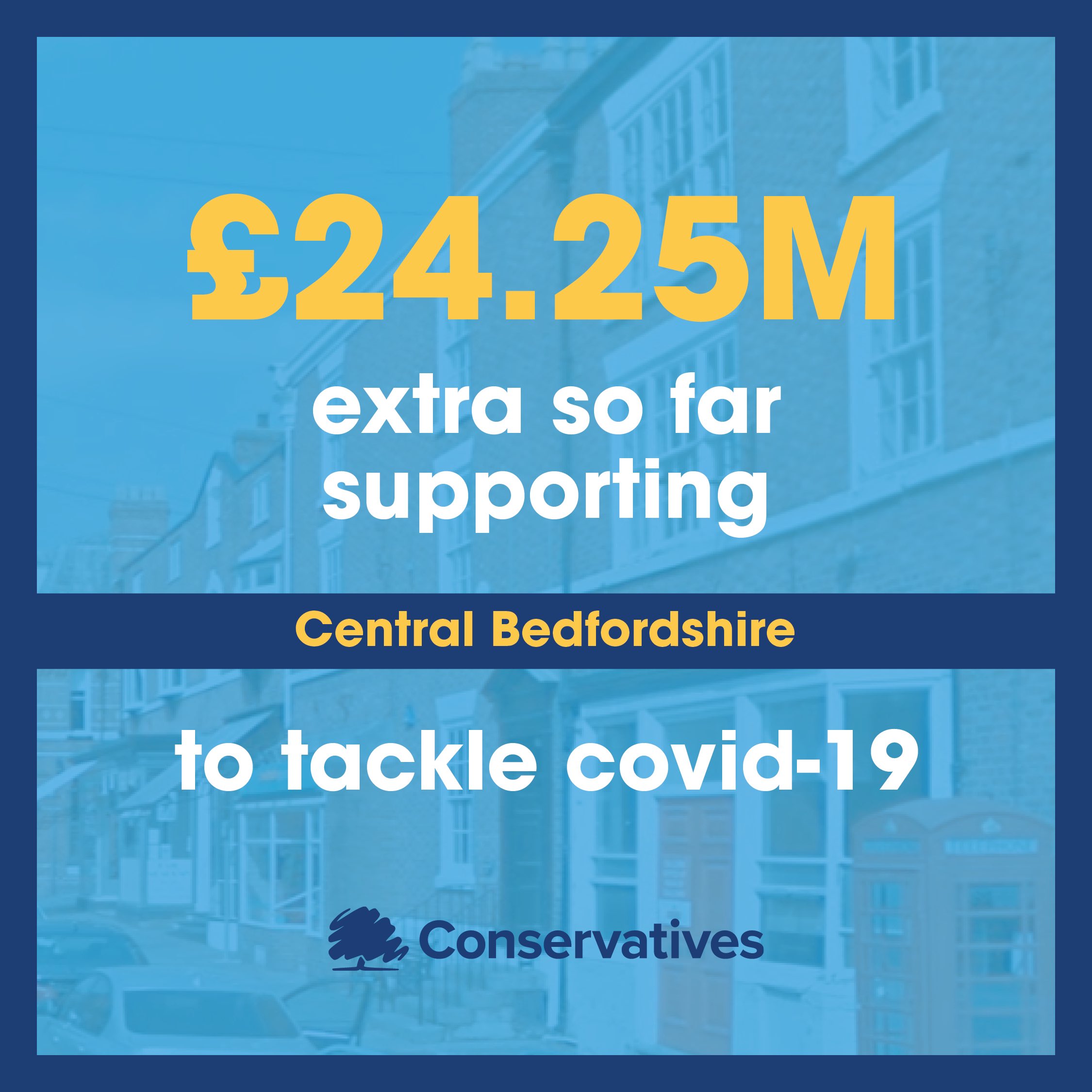
](https://twitter.com/NadineDorries/status/1319957105676308481/photo/1)

102 Last week the government provided councils with an Emergency Assistance Grant for Food and Essential Supplies - [http://GOV.UK](https://t.co/CxPKLIJmsu?amp=1) My own council ⁦

[@letstalkcentral](https://twitter.com/letstalkcentral)

⁩ received £ 217,555.3 to help with food supplies for disadvantaged families (downward arrow emoji)

https://twitter.com/NadineDorries/status/1319954925066067969?s=20

103 In addition to the money received to help with food supplies for disadvantaged families, on Friday we announced that

[@letstalkcentral](https://twitter.com/letstalkcentral)

will receive a further £2.5million not ring-fenced to help with general local [#COVID19](https://twitter.com/hashtag/COVID19?src=hashtag_click) local support.

https://twitter.com/NadineDorries/status/1319955565104320512?s=20

104 With no reliable vaccine, no understanding of how long immunity lasts or anti bodies are present and whilst reports of people infected a second time increase in numbers, when it comes to [#coronavirus](https://twitter.com/hashtag/coronavirus?src=hashtag_click) (until we have evidence to the contrary) there’s no such thing as herd immunity

https://twitter.com/NadineDorries/status/1319328041735172097?s=20

105 Closing the general [#Covid](https://twitter.com/hashtag/Covid?src=hashtag_click) debate today. The young are now helping to slow the spread of the virus by complying with social distancing rules. [#HandsFaceSpace](https://twitter.com/hashtag/HandsFaceSpace?src=hashtag_click)

https://twitter.com/NadineDorries/status/1319243553952960514?s=20

106 It’s the over 60s who are at risk. How do we shield 13m people?

https://twitter.com/NadineDorries/status/1318305174566412289?s=20

107 We are right now testing more people for [#coronavirus](https://twitter.com/hashtag/coronavirus?src=hashtag_click) than any other country in Europe. By the end of Oct, we will be up to 500,000 per day. Who needs a logo? The numbers speak for themselves.

https://twitter.com/NadineDorries/status/1318298236810121219?s=20

108 Middle of a warm October and rise in deaths is already above our winter Reasonable Worst Case scenario - deaths lag hospitalisations-lag cases. A nurse at Royal Liverpool ICU said, ‘we’re at full pressure and it’s not even winter yet, what are we going to do when we are?

https://twitter.com/NadineDorries/status/1317900081660645376?s=20

109 www.ozdic.com/ I've crunched the numbers and a herd immunity strategy would lead to a lot of deaths

https://twitter.com/NadineDorries/status/1317896159046868997?s=20

110 Sad to hear Andy Burnham on [#Marr](https://twitter.com/hashtag/Marr?src=hashtag_click) misrepresenting regions with low rates of infection right now. The rate in Cornwall, Devon and other areas is circa 30+ per 100.000. In Manchester it’s circa 400+ per 100,000.

https://twitter.com/NadineDorries/status/1317749804680826880?s=20

111 In the Royal an ICU nurse working a 12hr shift in full PPE will be watching this. Those revellers, will stagger home to their mams and dads, who will visit their nana and grandads. In a few days, they will think that nurse is an angel as she works a punishing shift to save a life

https://twitter.com/NadineDorries/status/1316457528545472512?s=20

112 Some have taken exception to my herd immunity comment. SoS had more than 140 characters and explains it much better here. Coronavirus: Matt Hancock rubbishes herd immunity | UK News | Sky News

https://twitter.com/NadineDorries/status/1316388766253281281?s=20

113 There are no words to describe how dangerous or depressing this is. [#COVID19](https://twitter.com/hashtag/COVID19?src=hashtag_click) [#Covid](https://twitter.com/hashtag/Covid?src=hashtag_click)

Quote Tweet

https://twitter.com/NadineDorries/status/1316339495181209601?s=20

114 And, then what?

https://twitter.com/NadineDorries/status/1316087710889775104?s=20

115 If herd immunity existed, measles and chicken pox would have been wiped out years ago. There is no such thing as herd immunity.

https://twitter.com/NadineDorries/status/1316086032908070926?s=20

116 You need to speak to your Manchester Mayor

[@AndyBurnhamGM](https://twitter.com/AndyBurnhamGM)

who has been opposed to intervention measures all the way!

Quote Tweet

https://twitter.com/NadineDorries/status/1316059017605795840?s=20

117 Fairy tale scenario of shield the elderly and let young go free, does not exist. At risk=over 60. Infection in the young absolutely transmits upwards. People over 60 cannot live in incubators. In our society many people live alone, they need to eat, visit Dr, live. [#Covid](https://twitter.com/hashtag/Covid?src=hashtag_click)

https://twitter.com/NadineDorries/status/1315924192089591809?s=20

118 Protect the [#NHS](https://twitter.com/hashtag/NHS?src=hashtag_click) is about ensuring we are never in the position of having no ICU beds available. People don’t just die in ICU, they are made better using cutting edge and emerging [#Covid](https://twitter.com/hashtag/Covid?src=hashtag_click) therapies. If the beds are full, where does the RTA go? Counting deaths is not the full story

https://twitter.com/NadineDorries/status/1315922817217634304?s=20

119 We know that even mild cases of [#Covid](https://twitter.com/hashtag/Covid?src=hashtag_click) can have devastating consequences with the risk of long Covid. [#HandsFaceSpace](https://twitter.com/hashtag/HandsFaceSpace?src=hashtag_click)

https://twitter.com/NadineDorries/status/1315677360721723398?s=20

120 Funding boost for Covid-19 mental health research | National Health Executive

https://twitter.com/NadineDorries/status/1315615907696316418?s=20

.

[@ITV](https://twitter.com/ITV)

Help Our Helplines has done an incredible job of highlighting the work of MH charities. Tonight,

[@antanddec](https://twitter.com/antanddec)

on [#BritainsGotTalent](https://twitter.com/hashtag/BritainsGotTalent?src=hashtag_click) will announce a Government donation of £1m to help the [#coronavirus](https://twitter.com/hashtag/coronavirus?src=hashtag_click) MH response fund administered by

[@MindCharity](https://twitter.com/MindCharity)

[#WorldMentalHealthDay](https://twitter.com/hashtag/WorldMentalHealthDay?src=hashtag_click)

https://twitter.com/NadineDorries/status/1314975861368594437?s=20

121 We don’t know wether or not we will need them. This is our first full winter living with [#COVID19](https://twitter.com/hashtag/COVID19?src=hashtag_click) They are there and we are prepared if we do.

https://twitter.com/NadineDorries/status/1314681568758247427?s=20

122 Only ventilated patients can be treated at the Nightingales. They are converted exhibition units, long lines of high tech beds, zero visitors or other facilities such as catering. They are for the most challenging of cases with patients asleep requiring the most intensive care.

Quote Tweet

https://twitter.com/NadineDorries/status/1314679394246496258?s=20

123 In recent weeks the highest rates of people testing positive for [#COVID](https://twitter.com/hashtag/COVID?src=hashtag_click)-19 are among older teenagers and young adults. This transfers to the older population following a lag which in turn follows through into hospital admissions. [https://ons.gov.uk/peoplepopulationandcommunity/healthandsocialcare/conditionsanddiseases/bulletins/coronaviruscovid19infectionsurveypilot/englandwalesandnorthernireland9october2020](https://t.co/8d1nJ6ntRq?amp=1)

https://twitter.com/NadineDorries/status/1314600105241124864?s=20

124 [#HandsFaceSpace](https://twitter.com/hashtag/HandsFaceSpace?src=hashtag_click)

https://twitter.com/NadineDorries/status/1314493972396617728?s=20

125 If you don’t believe the scientists, trust the doctors (emoji)

126 Get tough now to save the NHS from imploding, doctors urge

https://twitter.com/NadineDorries/status/1314461940081725445?s=20

127 They were and are. First thing we did was to draw up a local management outbreak plan with every LA CEO, Leader and DPH. Discussions take place daily

https://twitter.com/NadineDorries/status/1314304681506676756?s=20

128 The transmission rates tell a different story, Matt. Although I totally appreciate that many are and it must be especially frustrating for them.

https://twitter.com/NadineDorries/status/1314229390109102080?s=20

129 Young people may think they only need to self isolate/socially distance if they display symptoms and don’t appreciate that a huge amount of transmission occurs asymptomatically, when they won’t even know they have the virus. Follow the guidance at all times. [#HandsFaceSpace](https://twitter.com/hashtag/HandsFaceSpace?src=hashtag_click)

https://twitter.com/NadineDorries/status/1314223172150063110?s=20

130 Isolating older at risk (over 60s) looks good on paper,in reality it is not achievable. Infections are brought into care homes by care workers, into private homes by those who do their shopping or others with whom they live. Into hospitals by staff. No one can live in isolation

https://twitter.com/NadineDorries/status/1314142954420924416?s=20

[131 Over 60s have to eat, have their broken boiler fixed, see a Dr, talk to others. lonliness and isolation also kills.]( 131 Over 60s have to eat, have their broken boiler fixed, see a Dr, talk to others. lonliness and isolation also kills.https://twitter.com/NadineDorries/status/1314143112424566785?s=20)

[https://twitter.com/NadineDorries/status/1314143112424566785?s=20]( 131 Over 60s have to eat, have their broken boiler fixed, see a Dr, talk to others. lonliness and isolation also kills.https://twitter.com/NadineDorries/status/1314143112424566785?s=20)

132 Those who now claim that further measures are not needed, will in about ten days from now, when hospital admissions are at a critical stage argue that we didn’t do enough. We must do all we can to prevent our ICUs [#NHS](https://twitter.com/hashtag/NHS?src=hashtag_click) from becoming overwhelmed [#COVID19](https://twitter.com/hashtag/COVID19?src=hashtag_click)

https://twitter.com/NadineDorries/status/1314129698721542146?s=20

133 For those still claiming that face coverings don’t work in halting the transmission

Oxford COVID-19 study: face masks and coverings work – act now | University of Oxford

https://twitter.com/NadineDorries/status/1312329957541122050?s=20

134 I wish ⁦

[@realDonaldTrump](https://twitter.com/realDonaldTrump)

⁩ a good recovery from this ghastly [#Covid](https://twitter.com/hashtag/Covid?src=hashtag_click) virus. Trump flown to hospital

https://twitter.com/NadineDorries/status/1312300967304597505?s=20

135 She has been in the chamber with MPs for hours. In the corridors, offices, tea rooms, on long distance public travel. She has literally, knowingly put peoples lives at risk. At exactly what time did you know she was feeling unwell and had taken a test? [#COVID19](https://twitter.com/hashtag/COVID19?src=hashtag_click)

Quote Tweet

https://twitter.com/NadineDorries/status/1311752492435361792?s=20

136 At what time did

[@NicolaSturgeon](https://twitter.com/NicolaSturgeon)

know? Today is Thursday, when did contact tracing commence with everyone she has been in contact with? Why has it only been made public right now? Have staff in the hotel she stays been informed?

https://twitter.com/NadineDorries/status/1311753147954745345?s=20

137 We do have to live with it, Ruth, but not only ‘it.’ When all the ICU beds are taken with [#COVID19](https://twitter.com/hashtag/COVID19?src=hashtag_click) patients, our responsibility is to ensure the RTA or cardiac arrest pt has somewhere to be treated. We can’t let the virus spiral out of control and consume our [#NHS](https://twitter.com/hashtag/NHS?src=hashtag_click) services

Quote Tweet

https://twitter.com/NadineDorries/status/1311652023469776896?s=20

ابدأ من تويتات شهر 9 2020

138 Watching the [#ShipmanFiles](https://twitter.com/hashtag/ShipmanFiles?src=hashtag_click) [#BBC2](https://twitter.com/hashtag/BBC2?src=hashtag_click) His legacy is still reverberating in patient safety policy circles. Not heard it mentioned once yet, that the majority of his victims were women. Do hope this highlighted before the end of the program.


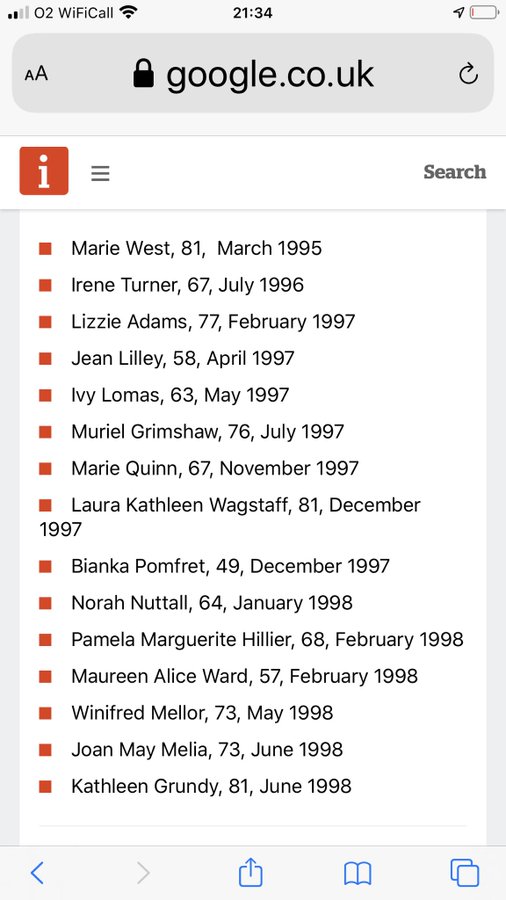


https://twitter.com/NadineDorries/status/1311404766925389832?s=20

139 Important to note, they do not die as a result of childbirth itself, but of complications due to general health issues such as diabetes. I am holding a second round table on this issue in Oct to probe further for answers and

[@TeamCMidO](https://twitter.com/TeamCMidO)

has done fantastic mitigating work on this

Quote Tweet

https://twitter.com/NadineDorries/status/1311321296983252992?s=20

140 The impact of pandemic on those with pre-existing mental health conditions could be significant. Great to see that as result of our £27m funding to see that ⁦

[@ThinkAheadMH](https://twitter.com/ThinkAheadMH)

⁩ . Applications for MH social workers are now open

https://twitter.com/NadineDorries/status/1311275542742798338?s=20

141 Since day 1 Labour MPs and elected mayors have sought to score points/play politics with [#COVID19](https://twitter.com/hashtag/COVID19?src=hashtag_click) - making complaints and then constantly shifting goal posts and being obstructive. Kate Greens only mistake was to let the cat out of the bag. Depressing

<https://twitter.com/NadineDorries/status/1308771020497727489?s=20>

142 Whilst passing legislation this week in committe, Labour politicians chose to filibuster and mount a whips operation, rather than engage in serious debate and discussion. I do not inc

[@leicesterliz](https://twitter.com/leicesterliz)

in this who is always constructive.

https://twitter.com/NadineDorries/status/1308771540121587713?s=20

143 You can tie the knot at a wedding and when these dreadful days are over, throw the big party and celebrate with friends and family. At a funeral, you only get one chance to pay your last respects and say goodbye.

https://twitter.com/NadineDorries/status/1308649861949067264?s=20

144 You can have a memorial service at a later date, but the body of the deceased will not be present. A memorial service is to reflect and remember, a funeral is to say goodbye.

https://twitter.com/NadineDorries/status/1308655844062629889?s=20

145 Grief is the last taboo and it is hard, so painful, especially if it has to be borne alone. I am delighted that ⁦at

[@DHSCgovuk](https://twitter.com/DHSCgovuk)

⁩ we have been able to provide funding to ensure that Cruse can meet the increased demand during these difficult days.

https://twitter.com/NadineDorries/status/1308460908365189121?s=20

146 If 30 kids are in the park feeding ducks, they will have 15 adults with them, in close proximity. 11 women playing netball, men playing football or any organised sport is very different. Wonder why the media only using grouse shooting as the sporting example? (emoji)

[#COVID19](https://twitter.com/hashtag/COVID19?src=hashtag_click)

https://twitter.com/NadineDorries/status/1308343460320210944?s=20

147 On it (thums up emoji) Jeremy [https://dailyecho.co.uk/news/18733931.amp/?__twitter_impression=true](https://t.co/ii7pPT5P3A?amp=1)

https://twitter.com/NadineDorries/status/1308104392541954048?s=20

148 Some are unhappy that we are hearing today from

[@CMO_England](https://twitter.com/CMO_England)

and the chief scientific officer today. We have always followed the science. The journalists have the opportunity to test that.

[@BorisJohnson](https://twitter.com/BorisJohnson)

will obviously address the nation this week as confirmed by

[@grantshapps](https://twitter.com/grantshapps)

https://twitter.com/NadineDorries/status/1307956520349569025?s=20

149 An amazing team ⁦

[@UHSFT](https://twitter.com/UHSFT)

⁩ who have launched their own Zero Covid campaign. Staff across the hospital community are working together to achieve this goal. Committed, determined and compassionate [#NHS](https://twitter.com/hashtag/NHS?src=hashtag_click) staff. Loved meeting you all. Keep up the good work!

https://twitter.com/NadineDorries/status/1307949379425370113?s=20

150 Patient safety is my [#No1](https://twitter.com/hashtag/No1?src=hashtag_click) priority and our healthcare workers are our [#No1](https://twitter.com/hashtag/No1?src=hashtag_click) asset.

https://twitter.com/NadineDorries/status/1307604862759301120?s=20

151 This [#WorldPatientSafetyDay](https://twitter.com/hashtag/WorldPatientSafetyDay?src=hashtag_click), I was delighted to announce a further £8.7m fund to help hospitals move to digital prescribing reducing prescribing errors by up to 30%.

https://twitter.com/NadineDorries/status/1306872620135129089?s=20

152 Thank you to

[@UHSFT](https://twitter.com/UHSFT)

University Hospital Southampton for hosting us at your virtual [#WorldPatientSafetyDay](https://twitter.com/hashtag/WorldPatientSafetyDay?src=hashtag_click) event today, a wonderful opportunity to meet your hardworking staff !

https://twitter.com/NadineDorries/status/1306605180528594947?s=20

153 We all have to play our part and do everything we can to keep hospital admissions down. We can only do this by abiding by the guidance and socially distancing in order to protect our [#NHS](https://twitter.com/hashtag/NHS?src=hashtag_click) [#Ruleof6](https://twitter.com/hashtag/Ruleof6?src=hashtag_click) [#COVID19](https://twitter.com/hashtag/COVID19?src=hashtag_click)

https://twitter.com/NadineDorries/status/1305610639449231360?s=20

154 It’s possibly behavioural. In social settings people (usually) drink alcohol, are happy to see each other, excited, chatty and social distancing adherence may be temporarily forgotten. In the workplace and on public transport, alcohol consumption rare! [#Covid19](https://twitter.com/hashtag/Covid19?src=hashtag_click)

https://twitter.com/NadineDorries/status/1303998117927366656?s=20

155 Only apply for a [#Covid19](https://twitter.com/hashtag/Covid19?src=hashtag_click) test if you have symptoms * High temperature * New or continuous cough * Change to taste or smell An increase in asymptomatic people (no symptoms) applying for tests creates a delay/ inconvenience for people who are ill

https://twitter.com/NadineDorries/status/1303607950284595200?s=20

156 So grateful to Davina and others for making the time to do this. The effects of the pandemic on children & young people’s mental health have been challenging. It’s vital we do all we can to protect them to prevent any long-term impact [#EveryMindMatters](https://twitter.com/hashtag/EveryMindMatters?src=hashtag_click) [https://uk.news.yahoo.com/davina-mccall-marvin-humes-launch-110741689.html](https://t.co/sy7ywXajjZ?amp=1)

https://twitter.com/NadineDorries/status/1303318620789968898?s=20

157 No one who knew anything about the pathology of [#Covid19](https://twitter.com/hashtag/Covid19?src=hashtag_click) ever said it would be over by Christmas.

Quote Tweet

https://twitter.com/NadineDorries/status/1303235825057046528?s=20

158 There is a time lag, Toby. We have more knowledge regarding the the pathology of this particularly nasty [#coronavirus](https://twitter.com/hashtag/coronavirus?src=hashtag_click) It likes cooler temperatures, they have gone one way (upward triangle emoji)

159 We know it transfers from the more mobile age groups to the more vulnerable and ...the uptick moves (upward triangle emoji)

<https://twitter.com/toadmeister/status/1302697085947568128>

https://twitter.com/NadineDorries/status/1302705852412047364?s=20

160 We can reverse this, [#Handwashing](https://twitter.com/hashtag/Handwashing?src=hashtag_click) [#SocialDistancing](https://twitter.com/hashtag/SocialDistancing?src=hashtag_click) [#handsfacespace](https://twitter.com/hashtag/handsfacespace?src=hashtag_click) [#WearAMask](https://twitter.com/hashtag/WearAMask?src=hashtag_click)

[
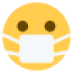
](https://twitter.com/hashtag/WearAMask?src=hashtag_click)

161 We know that a rise in the younger age groups will transfer across to those who are more at risk and vulnerable. Keep your Nan and grandad safe. (old woman emoji) (old man emoji)

[#COVID19](https://twitter.com/hashtag/COVID19?src=hashtag_click) [#coronavirus](https://twitter.com/hashtag/coronavirus?src=hashtag_click)

https://twitter.com/NadineDorries/status/1302689104732266500?s=20

162 Thank you Sean, and your team for your collaborative approach and your determination to beat [#Covid](https://twitter.com/hashtag/Covid?src=hashtag_click) in Oldham.

https://twitter.com/NadineDorries/status/1296389445353123840?s=20

163 Today we are launching an £8m Wellbeing for Education Return programme for schools/college to help parents/staff spot at an early stage when children are struggling through the negative effects of [#Covid](https://twitter.com/hashtag/Covid?src=hashtag_click) and enable them to offer MH and emotional support

https://twitter.com/NadineDorries/status/1295250039745519616?s=20

164 The effects of [#Covid](https://twitter.com/hashtag/Covid?src=hashtag_click) on many people’s mental health and wellbeing may be long lasting. The NHS, local services and Government funded charities have been there to help throughout. It is important we now ensure our services meet any rise in demand.

https://twitter.com/NadineDorries/status/1294583319456292864?s=20

165 Best wishes to all A level students in MidBeds and everywhere. This pandemic has been so hard. Take heart, my daughter didn’t get the grades, the uni or the course she wanted. Went into clearing and graduated with a 1st in law. This is your time - so many opportunities ahead.

https://twitter.com/NadineDorries/status/1293875242725777408?s=20

166 Our strategy of managing regional outbreaks in order to protect the rest of the U.K. has been praised by WHO chief ⁦

[@DrTedros](https://twitter.com/DrTedros)

⁩ WHO chief praises northern England lockdown as global Covid-19 cases edge towards 20 million

https://twitter.com/NadineDorries/status/1292869681662042112?s=20


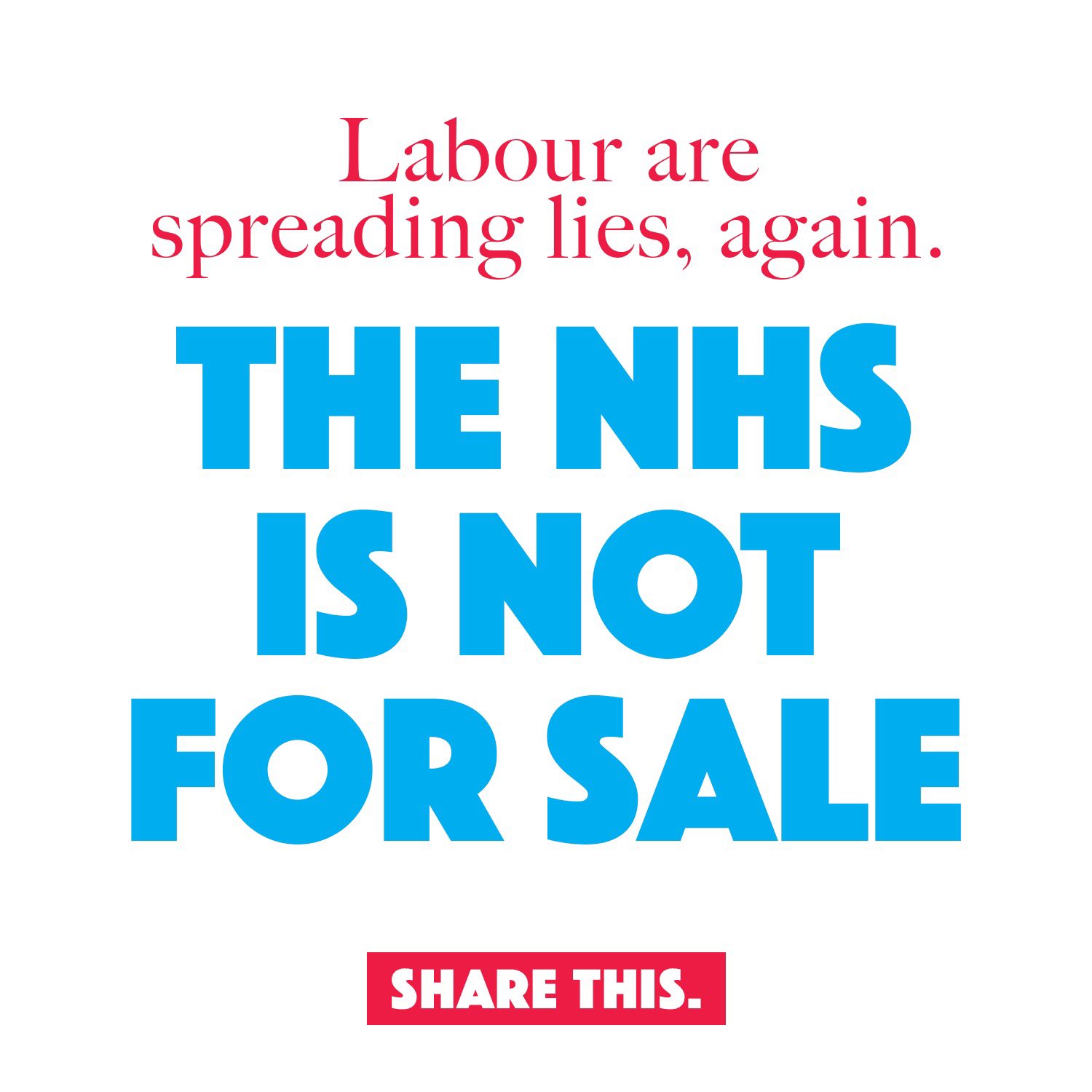
https://twitter.com/NadineDorries/status/1286028158458908681?s=20

167 Leading the world (British flag emoji)

[#Covid19](https://twitter.com/hashtag/Covid19?src=hashtag_click) [#Vaccine](https://twitter.com/hashtag/Vaccine?src=hashtag_click)

Quote Tweet

https://twitter.com/NadineDorries/status/1285319481875730433?s=20

168 Merkel and Macron walked out of the room- Italy, Spain angry as EU leaders fail to agree on how to divvy up the money. In the U.K. funding was announced and delivered weeks ago 750bn coronavirus rescue fund in chaos as EU squabbles over purse strings

https://twitter.com/NadineDorries/status/1285105552767553537?s=20

169 So annoying, last week I could taste coffee, today I can’t. A fruit salad on Thursday was delicious. Yesterday, a peach tasted like battery acid. [#Ageusia](https://twitter.com/hashtag/Ageusia?src=hashtag_click) [#COVID19](https://twitter.com/hashtag/COVID19?src=hashtag_click) [#Thethingswedontyetknow](https://twitter.com/hashtag/Thethingswedontyetknow?src=hashtag_click)

https://twitter.com/NadineDorries/status/1284375034065231872?s=20

170 Has anyone told him yet that he lost? This statement is completely untrue. A propoganda hangover from the Labour election campaign. (downward triangle emoji)

171 [#FakeNews](https://twitter.com/hashtag/FakeNews?src=hashtag_click)

https://twitter.com/NadineDorries/status/1284101283247542272?s=20

172 The

[@thetimes](https://twitter.com/thetimes)

front page on 17th March. The first line reads, ‘Britain was put on hold last night.’ That was the 16th March.


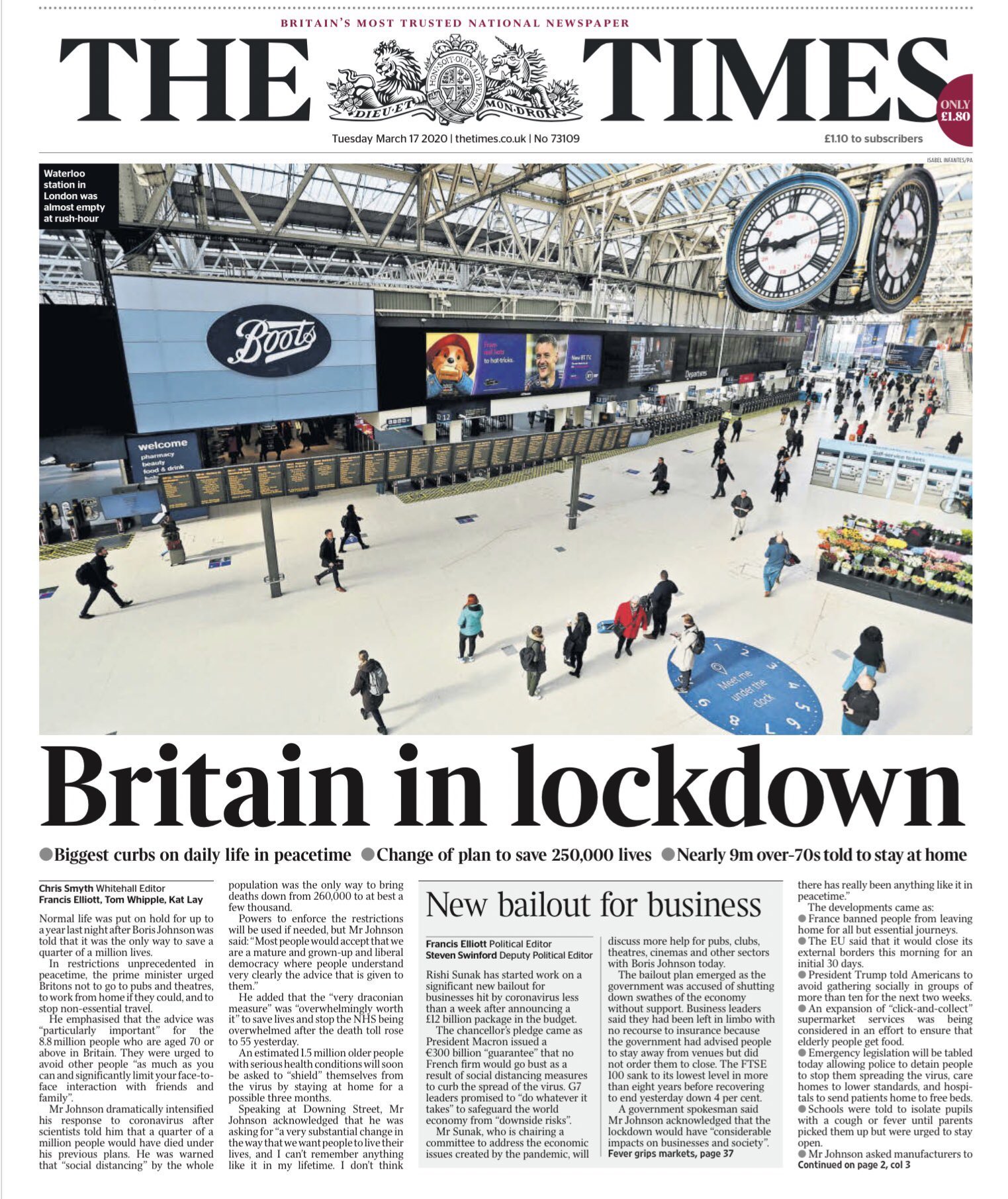


https://twitter.com/NadineDorries/status/1284050414619459586?s=20

173 The area of Leicester in lockdown has a [#Covid19](https://twitter.com/hashtag/Covid19?src=hashtag_click) positivity rate three times higher than the next highest area in the U.K. These measures are necessary to keep Leicester and all of us safe.

https://twitter.com/NadineDorries/status/1284015086890352640?s=20

174 Being a Minister ⁦

[@campbellclaret](https://twitter.com/campbellclaret)

⁩ is long hours spent behind a desk making tough decisions, not in front of a camera. Try NO reduction in MH services during pandemic. New 24hr crisis helplines across U.K. £5 million given to ⁦

[@MindCharity](https://twitter.com/MindCharity)

⁩

https://twitter.com/NadineDorries/status/1283306488610652160?s=20

175 Funding charities and helping those most affected by lockdown -BEAT helping YP eating disorders/bereavement/Every Mind Matters. We’ve also been fighting [#COVID19](https://twitter.com/hashtag/COVID19?src=hashtag_click) in

[@DHSCgovuk](https://twitter.com/DHSCgovuk)

You keep on courting publicity, attempting to remain relevant, I’ll carry on working.

https://twitter.com/NadineDorries/status/1283308479093133312?s=20

176 Replying to

[@ianbirrell](https://twitter.com/ianbirrell)

[@Penbat1](https://twitter.com/Penbat1)

and

[@MattHancock](https://twitter.com/MattHancock)

Absolutely untrue Ian. The work on patient safety is relentless and features at the top of my agenda daily. But you wouldn’t know that or have a clue about what is achieved, but free to comment - let Twitter be your guiding star to the fount of no knowledge and certain opinion.

https://twitter.com/NadineDorries/status/1283014605372043264?s=20

177 England has lowest rate of COVID-19 care home deaths, European survey

https://twitter.com/NadineDorries/status/1281159087770734592?s=20

178 Being looked at, but no one knew. I’ve never met anyone, ever who had the flu or a head old who told me they were asymptomatic. Asymptomatic transmission is another arrow in [#COVID19](https://twitter.com/hashtag/COVID19?src=hashtag_click) ‘s quiver which has made this virus so difficult to deal with.

https://twitter.com/NadineDorries/status/1280850309657608194?s=20

179 Even if you have had [#Covid19](https://twitter.com/hashtag/Covid19?src=hashtag_click) you must continue to protect yourself and others. Coronavirus antibody protection may only last weeks

https://twitter.com/NadineDorries/status/1280391355299225600?s=20

180 Decision is down to individual trusts and what they deem to be safe. As you may know, some scan rooms are small, windowless and social distancing with equipment could be difficult. Safety for all, inc [#NHS](https://twitter.com/hashtag/NHS?src=hashtag_click) staff is paramount - many services being reviewed

https://twitter.com/NadineDorries/status/1279385912020029440?s=20

181 (explosion emoji)
[#COVID19](https://twitter.com/hashtag/COVID19?src=hashtag_click)

https://twitter.com/NadineDorries/status/1278659678319378435?s=20

182 For almost two weeks, the people of Leicester looked for local leadership and guidance. They heard and saw the city mayor on radio and television stating that he didn’t believe the data Stay at home Leicester as much as possible and nail this [#Covid19](https://twitter.com/hashtag/Covid19?src=hashtag_click)

https://twitter.com/NadineDorries/status/1278577530975465472?s=20

183 Leicester boundary decision will be taken by local council leaders working with DPH and others on the ground who know Leicester street by street - using infection rate information provided by the centre. This is the essence of how local outbreak management plans will work.

https://twitter.com/NadineDorries/status/1277862880608649217?s=20

184 Well done to

[@MyDoncaster](https://twitter.com/MyDoncaster)

If there was a prize for LA T&T comms, they’ve already won it. [#COVID19](https://twitter.com/hashtag/COVID19?src=hashtag_click)

https://twitter.com/NadineDorries/status/1277274962487894017?s=20

185 Tackling health inequalities ensuring BAME women get the right support during pregnancy is one of my top priorities As we work rapidly to understand risk factors, it is vital Trusts take all possible steps to minimise the risk of COVID-19 for BAME women

https://twitter.com/NadineDorries/status/1276823329035476992?s=20

186

The ⁦

[@WHO](https://twitter.com/WHO)

⁩ has congratulated Britain for doing well. Now it’s our job to accept that the virus is amongst us and we must all exercise our common sense in order to keep ourselves and everyone else safe.

https://twitter.com/NadineDorries/status/1275713624108122112?s=20

187 And the ⁦

[@NursingTimes](https://twitter.com/NursingTimes)

⁩ covers yesterday’s announcement - Nurses offered 'psychological first aid' training tailored for Covid-19 | Nursing Times

https://twitter.com/NadineDorries/status/1272775096822833154?s=20

188 Today’s ⁦⁦ Northamptonshire | Daventry Express reports that Coronavirus test and trace services get £3m boost - all upper tier authorities have been given funding to help manage local outbreaks ⁦

[@DxGusher](https://twitter.com/DxGusher)

https://twitter.com/NadineDorries/status/1272774324479569920?s=20

189 Delighted to announce a new

[@PHE_uk](https://twitter.com/PHE_uk)

[#PsychologicalFirstAid](https://twitter.com/hashtag/PsychologicalFirstAid?src=hashtag_click) Training course today, open to all those working to support those most in need both through the coronavirus outbreak and beyond: [https://gov.uk/government/news/psychological-first-aid-in-emergencies-training-for-frontline-staff-and-volunteers…](https://t.co/nwbMMcoD3F?amp=1)

https://twitter.com/NadineDorries/status/1272556927462113280?s=20

190 Full scale lockdown is what test/trace +local outbreak management is there avoid and to prevent R from rising and virus spreading. The Chancellor himself this morning made clear that a second peak would be disastrous for our economy so we need to do what’s necessary to avoid that

https://twitter.com/NadineDorries/status/1272132537578655745?s=20

191 We have given each council what it needs to develop local outbreak management plans because we are serious about beating [#Covid19](https://twitter.com/hashtag/Covid19?src=hashtag_click) Essex County Council gets £5.7m to help NHS ‘test and trace’ | Clacton and Frinton Gazette [https://gazette-news.co.uk/news/north_essex_news/18514290.essex-county-council-gets-5-7m-help-nhs-test-trace/](https://t.co/2yV77uzMOi?amp=1)

https://twitter.com/NadineDorries/status/1271523405121060864?s=20

192 It is such a huge privilege and pleasure to work with local authorities in the development of their local outbreak management plans. Ensuring adequate funds are in place to enable LAs to hunt down the virus was key to this objective. [#Covid19](https://twitter.com/hashtag/Covid19?src=hashtag_click)

https://twitter.com/NadineDorries/status/1271384838650675201?s=20

193 We cannot bend the social distancing rules. We cannot get our children back into school due to the size of our existing school buildings. If we could, we would. [#Covid19](https://twitter.com/hashtag/Covid19?src=hashtag_click)

https://twitter.com/NadineDorries/status/1270764985531215873?s=20

194 If you are living with an eating disorder, help us there for you during [#Covid19](https://twitter.com/hashtag/Covid19?src=hashtag_click)

https://twitter.com/NadineDorries/status/1270672432379187200?s=20

195 Extra mental health support for pupils and teachers. Investing in and utilising new technology to help pupils and teachers in our schools. [#Covid19](https://twitter.com/hashtag/Covid19?src=hashtag_click) has shone a light on importance of looking after mental health, especially our children and young people.

https://twitter.com/NadineDorries/status/1269526191112847361?s=20

196 Whilst having every sympathy, I’m struggling to see how, if all of these demonstrations do take place and many 1000s of people across the country break social distancing, how we keep R<1 and avoid reverting into full national lockdown again

https://twitter.com/NadineDorries/status/1268668221504315396?s=20

197 This is so true. We have the capacity and the ability, but we need people to come forward for testing in order to do the tests. We can’t force them on people!

https://twitter.com/NadineDorries/status/1268555848638480385?s=20

198 Delighted she’s not working, we’re on top of [#COVID19](https://twitter.com/hashtag/COVID19?src=hashtag_click) and it’s going the right way. More worrying if all 25,000 contact tracers were working flat out, now that would be a story. 99% of tracers delighted to be part of the resilience effort and doing a great job (thumbs up emoji)

https://twitter.com/NadineDorries/status/1268133955439599619?s=20

199 Fascinating to watch this and the speed at which [#Covid19](https://twitter.com/hashtag/Covid19?src=hashtag_click) transmits with fatal results. Global Deaths Due to Various Causes and COVID-19 | Flourish

https://twitter.com/NadineDorries/status/1267850151655280642?s=20

200 ‘For that reason, we will return to the taxpayer the funds we took from government to finance our furlough scheme and withdraw from that scheme forthwith.’ A fine example. Well done

[@spectator](https://twitter.com/spectator)

and

[@afneil](https://twitter.com/afneil)

[#InThisTogether](https://twitter.com/hashtag/InThisTogether?src=hashtag_click) [#Covid19](https://twitter.com/hashtag/Covid19?src=hashtag_click)

https://twitter.com/NadineDorries/status/1267721884382007297?s=20

201 Great work, Nina. (Collision symbol emoji) + (microbe emoji)

Quote Tweet

https://twitter.com/NadineDorries/status/1267135687301771264?s=20

202 Behind negative media/ criticism, good people are working day and night to beat [#Covid19](https://twitter.com/hashtag/Covid19?src=hashtag_click) Thousands across U.K. from scientists, Drs, biochemists, academics, CSs to contract tracers. When you attack, you demoralise and undermine their huge effort. Thank you David [#InThisTogether](https://twitter.com/hashtag/InThisTogether?src=hashtag_click)

https://twitter.com/NadineDorries/status/1266745040677810177?s=20

203 The police have made clear they are taking no action against Mr Cummings over his self-isolation and that going to Durham did not breach the regulations. Case closed. Let’s now focus on how we get out of lockdown and support [#TestTrackAndTrace](https://twitter.com/hashtag/TestTrackAndTrace?src=hashtag_click) [#COVID19](https://twitter.com/hashtag/COVID19?src=hashtag_click) [#InThisTogether](https://twitter.com/hashtag/InThisTogether?src=hashtag_click)

https://twitter.com/NadineDorries/status/1265988612988043264?s=20

204 Also told you, as did I, that local councils already have a plan in place to deal with outbreaks of local infections and that whilst they worked on developing local outbreak management plans for [#COVID19](https://twitter.com/hashtag/COVID19?src=hashtag_click) they would be deployed. Disappointing misinterpretation. [#InThisTogether](https://twitter.com/hashtag/InThisTogether?src=hashtag_click)

https://twitter.com/NadineDorries/status/1265958016257609729?s=20

205 Time to turn tables on [#COVID19](https://twitter.com/hashtag/COVID19?src=hashtag_click) If you have symptoms, you will be tested. If + we will trace your recent contacts who will be asked to self isolate for 14days. Local outbreaks will be handled by LAs to prevent a further national lockdown. We’re coming after you, [#coronavirus](https://twitter.com/hashtag/coronavirus?src=hashtag_click)


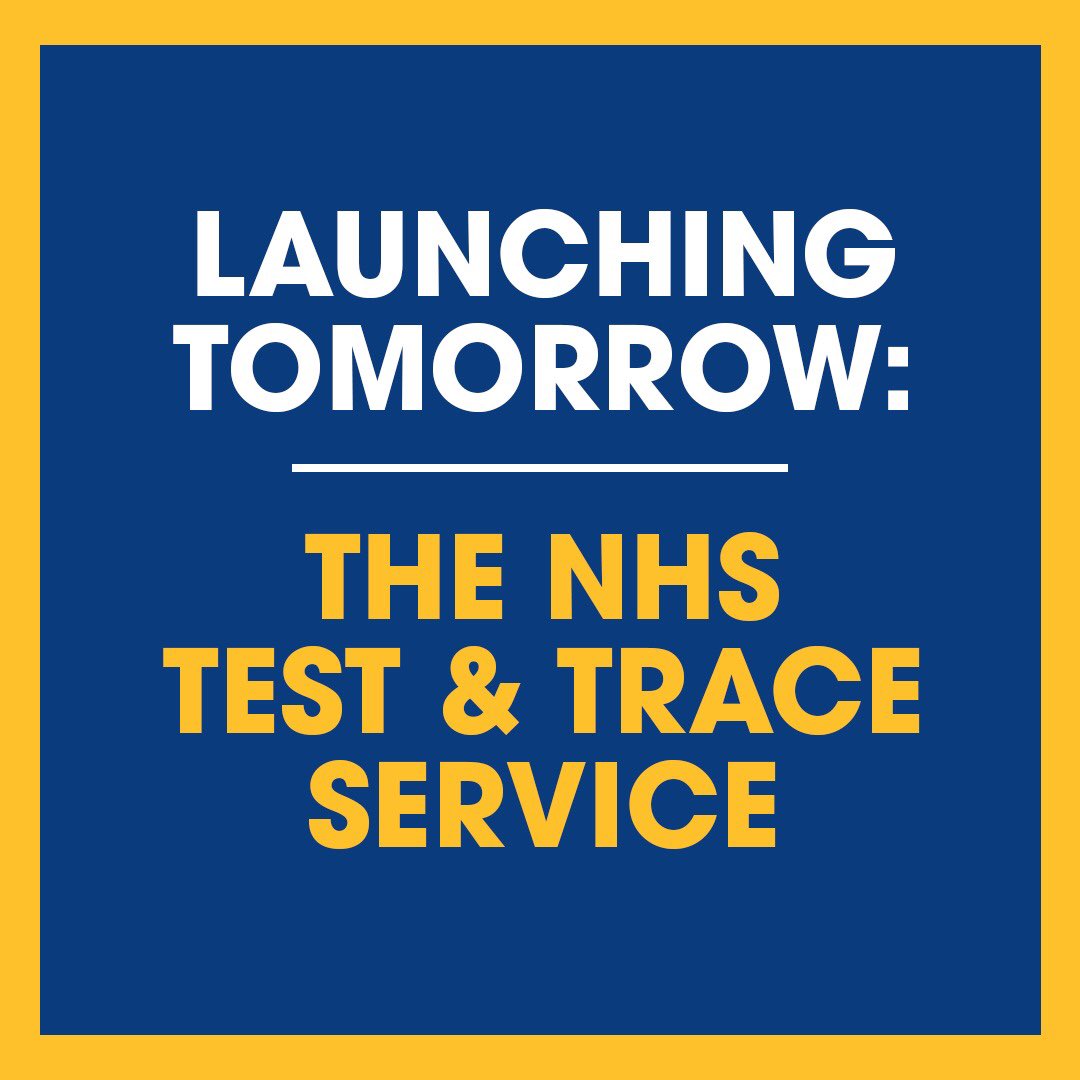


https://twitter.com/NadineDorries/status/1265729332951089152?s=20

206 Whatever your position on Cummings is, I think most sensible people can agree, the harassment of his wife and 4yo child alone in the house, baying media, loud and aggressive abuse, threats of violence is inhumane. Let’s get on with fighting [#Covid19](https://twitter.com/hashtag/Covid19?src=hashtag_click) not terrifying a small child

https://twitter.com/NadineDorries/status/1265310528047644672?s=20

207 Just finished a zoom call with our ten beacon councils and new advisory group who will be leading the way in the battle against [#Covid19](https://twitter.com/hashtag/Covid19?src=hashtag_click) at a local level. [#WhackTheMole](https://twitter.com/hashtag/WhackTheMole?src=hashtag_click) £300 million additional funding for local authorities to support new test and trace

https://twitter.com/NadineDorries/status/1263854859075358720?s=20

208 Lovely to see them so happy, but just a small point, when they pushed the mask down to drink the coffee, if there had been virus on the outside, it’s now all over their fingers and the coffee cup. [#Don](https://twitter.com/hashtag/Don?src=hashtag_click)’tTouchTheMask [#Covid19](https://twitter.com/hashtag/Covid19?src=hashtag_click)

https://twitter.com/NadineDorries/status/1263809272388947969?s=20

209 Yesterday I helped out with the

[@NHSEngland](https://twitter.com/NHSEngland)

Volunteer Responder scheme by chatting with Rita to see how she's doing in lockdown. Now more than ever it’s important we check in on those living alone to improve wellbeing [#MentalHealthAwarenessWeek](https://twitter.com/hashtag/MentalHealthAwarenessWeek?src=hashtag_click)

https://twitter.com/NadineDorries/status/1263739605834256385?s=20

210 We’ve just announced £4.2m of additional funding to mental health charities providing vital support to people affected by the COVID-19 pandemic.This is in addition to £5m to

[@MindCharity](https://twitter.com/MindCharity)

and £2.3billion overall allocated to the Mental Health by the PM [#MentalHealthAwarenessWeek](https://twitter.com/hashtag/MentalHealthAwarenessWeek?src=hashtag_click)

https://twitter.com/NadineDorries/status/1263502788035821568?s=20

211 My interview with ⁦

[@itvnews](https://twitter.com/itvnews)

⁩ yesterday on the fantastic provision ⁦the Government via ⁦

[@NHSuk](https://twitter.com/NHSuk)

⁩ have put in place for front line workers during the pandemic | Central - ITV News

https://twitter.com/NadineDorries/status/1263365447401029633?s=20

212 .

[@davidnabarro](https://twitter.com/davidnabarro)

from

[@WHO](https://twitter.com/WHO)

also said that if schools put appropriate and recommended measures in place, it’s safe for children to phase return to school, his own grandchildren too. I’m really surprised Sky haven’t tweeted that part of the interview out. (thinking face emoji)

https://twitter.com/NadineDorries/status/1262650262650523652?s=20

213 I can only agree with this. Today was a ‘Corona day.’ Breathless, tight chest, fatigue, can’t speak in consecutive sentences, vivid nightmares.The last one was four days ago when I staggered from work desk to bed at 8pm. It’s the weirdest thing. [#Covid19](https://twitter.com/hashtag/Covid19?src=hashtag_click)

Quote Tweet

https://twitter.com/NadineDorries/status/1261756748010082305?s=20

214 Never a believer in the nanny state, but the fact that obesity is the second biggest coronavirus risk factor and 25% of Brits are obese, has to be a wake up call. It’s time for us all to get on our bikes. [#COVID19](https://twitter.com/hashtag/COVID19?src=hashtag_click)

https://twitter.com/NadineDorries/status/1261207683161563136?s=20

215 I’m not sure who the ‘experts’ are quoted in this article stating that [#COVID19](https://twitter.com/hashtag/COVID19?src=hashtag_click) antibodies may last 2/3 years. We have no idea yet if they even last 2/3 months, or how effective they are. They really should cite the evidence and name the experts.

Quote Tweet

https://twitter.com/NadineDorries/status/1260677439849627653?s=20

216 .[#HappyInternationalNurseDay](https://twitter.com/hashtag/HappyInternationalNurseDay?src=hashtag_click) Never all those years ago could I have imagined what our nurses today have to deal with on a day to day basis. We are so grateful, thank you. [#Covid19](https://twitter.com/hashtag/Covid19?src=hashtag_click)

https://twitter.com/NadineDorries/status/1260264767027122176?s=20

217 If you are wearing a [#facecovering](https://twitter.com/hashtag/facecovering?src=hashtag_click) in public, please do not touch the front to remove, or to dip it down to talk or answer a phone - if you do, you could spread the virus onto your fingers. Always remove from the ties behind the ears or from the rear. [#facecovering](https://twitter.com/hashtag/facecovering?src=hashtag_click)

<https://twitter.com/NadineDorries/status/1260105093518708737?s=20>

218 Care home statement: ‘At Lark Hill, we have over three months’ supply of Personal Protective Equipment, including over 25,000 pairs of gloves, 7,700 aprons and nearly 6,000 masks.’ Are you sure that speaking out was the reason she was sacked?

https://twitter.com/NadineDorries/status/1258144246542057480?s=20

219 An important thing to remember today, is that this disease is so new, we don’t yet know once someone has had [#COVID19](https://twitter.com/hashtag/COVID19?src=hashtag_click) how long antibodies are present to provide immunity and therefore, how long immunity lasts. [#StaySafe](https://twitter.com/hashtag/StaySafe?src=hashtag_click) [#InThisTogether](https://twitter.com/hashtag/InThisTogether?src=hashtag_click)

https://twitter.com/NadineDorries/status/1257945019467681792?s=20

220 Honoured and thrilled to have been promoted today by

[@BorisJohnson](https://twitter.com/BorisJohnson)

to Minister of State at

[@DHSCgovuk](https://twitter.com/DHSCgovuk)

where we are all working together in the battle against [#COVID19](https://twitter.com/hashtag/COVID19?src=hashtag_click) [#Inthistogether](https://twitter.com/hashtag/Inthistogether?src=hashtag_click) [#StayHomeSaveLives](https://twitter.com/hashtag/StayHomeSaveLives?src=hashtag_click)

https://twitter.com/NadineDorries/status/1257781391896477703?s=20

221 Happy International Day of the Midwife to all the amazing midwifes around the country who are working tirelessly to provide essentials services. We need your skills now more than ever, thank you. [#InternationalDayOfTheMidwife](https://twitter.com/hashtag/InternationalDayOfTheMidwife?src=hashtag_click) [#Covid19](https://twitter.com/hashtag/Covid19?src=hashtag_click) [https://drive.google.com/file/d/1jT8tZqiC3y3i9FPwIMlphSZc4iRiS2D3/view?ts=5eb16114](https://t.co/FdWPTN9Qg6?amp=1)

https://twitter.com/NadineDorries/status/1257671339864973314?s=20

222 The residents of the Isle of Wight are making history. Leading the test, track and trace strategy against [#COVID19](https://twitter.com/hashtag/COVID19?src=hashtag_click) Thank you to everyone who downloads the app.

Quote Tweet

https://twitter.com/NadineDorries/status/1257420474121949192?s=20

223 Well said that man.

[@MaajidNawaz](https://twitter.com/MaajidNawaz)

Quote Tweet

https://twitter.com/NadineDorries/status/1256940689620971520?s=20

224 When you remove it, do so from the elastic at the back of the ears. If you touch the front of the mask, you will spread the virus (if it’s there) onto your hands. For the same reason, don’t touch the mask to lower it to speak over and wash after every use..

Quote Tweet

https://twitter.com/NadineDorries/status/1256308520942743552?s=20

225 Ahem...

https://twitter.com/NadineDorries/status/1256278584160911365?s=20

226 This is an excellent graphic which explains very clearly why it is so important to keep R<1

Quote Tweet

https://twitter.com/NadineDorries/status/1255950653265981441?s=20

227 . [#RossKempNHS](https://twitter.com/hashtag/RossKempNHS?src=hashtag_click) is back at Milton Keynes Hospital. A fantastic hospital and staff dealing with [#Covid19](https://twitter.com/hashtag/Covid19?src=hashtag_click)

https://twitter.com/NadineDorries/status/1255944011069763593?s=20

228 Our fantastic

[@RoyalMail](https://twitter.com/RoyalMail)

who have played a huge role during [#COVID19](https://twitter.com/hashtag/COVID19?src=hashtag_click) have re painted and dedicated a post box in Mid Beds to Colonel Tom. It’s painted in [#NHS](https://twitter.com/hashtag/NHS?src=hashtag_click) blue and located at the Post Office in Marston Moretaine

229 Well done! [#HappyBirthdayCaptainTom](https://twitter.com/hashtag/HappyBirthdayCaptainTom?src=hashtag_click)

https://twitter.com/NadineDorries/status/1255834253444681729?s=20

230 I’m afraid that’s not the case. You can still carry [#COVID19](https://twitter.com/hashtag/COVID19?src=hashtag_click) If you do have antibodies, we don’t know how effective they are or how long they last. We don’t yet know if everyone develops antibodies. Please follow the guidance. [#StayAtHome](https://twitter.com/hashtag/StayAtHome?src=hashtag_click)

https://twitter.com/NadineDorries/status/1255829439860150279?s=20

231 Do you know someone who has passed and are experiencing the emotions associated with loss and grief? It’s difficult enough when you have time to prepare.... [#Grief](https://twitter.com/hashtag/Grief?src=hashtag_click) [#Covid19](https://twitter.com/hashtag/Covid19?src=hashtag_click) [#MentalHealth](https://twitter.com/hashtag/MentalHealth?src=hashtag_click)

https://twitter.com/NadineDorries/status/1255585808712716288?s=20

232 [#TopTips](https://twitter.com/hashtag/TopTips?src=hashtag_click) for looking after your mental health and well-being during [#lockdown](https://twitter.com/hashtag/lockdown?src=hashtag_click) [#COVID19](https://twitter.com/hashtag/COVID19?src=hashtag_click) [#EveryMindMatters](https://twitter.com/hashtag/EveryMindMatters?src=hashtag_click)

https://twitter.com/NadineDorries/status/1254786977724915712?s=20

233 Well, we never saw that coming. (woman facepalming)

https://twitter.com/NadineDorries/status/1254743695187992577?s=20

234 Most who have been as poorly as

[@BorisJohnson](https://twitter.com/BorisJohnson)

with [#Covid19](https://twitter.com/hashtag/Covid19?src=hashtag_click) and a patient for a week in intensive care, would be off work for least three months to fully recover their strength and repair their immune system. Our PM is back after just three weeks. Good luck boss.

https://twitter.com/NadineDorries/status/1254656323637121025?s=20

235 *Game Changer Alert* Fantastic to see our scientists & business community taking on challenge of [#COVID19](https://twitter.com/hashtag/COVID19?src=hashtag_click) developing high-quality solution that will hopefully meet our exacting regulatory standards. Good luck! The British people want a test they can trust.

https://twitter.com/NadineDorries/status/1254305538520887296?s=20
